# Efficacy of Various Complexing Agents for Displacing Biologically Important Ligands from Eu(III) and Cm(III) Complexes in Artificial Body Fluids—An In Vitro Decorporation Study

**DOI:** 10.3390/ijms26157112

**Published:** 2025-07-23

**Authors:** Sebastian Friedrich, Antoine Barberon, Ahmadabdurahman Shamoun, Björn Drobot, Katharina Müller, Thorsten Stumpf, Jerome Kretzschmar, Astrid Barkleit

**Affiliations:** 1 Helmholtz-Zentrum Dresden-Rossendorf, Institute of Resource Ecology, 01328 Dresden, Germany; s.friedrich@hzdr.de (S.F.); b.drobot@hzdr.de (B.D.); k.mueller@hzdr.de (K.M.); t.stumpf@hzdr.de (T.S.); j.kretzschmar@hzdr.de (J.K.); 2 National School of Chemistry Montpellier, 34090 Montpellier, France; antoine.barberon@enscm.fr; 3 Institute of Radioecology and Radiation Protection, Leibniz University Hannover, 30419 Hannover, Germany; shamoun@irs.uni-hannover.de

**Keywords:** *f*-elements, trivalent actinides and lanthanides, chelating agents, aminopolycarboxylates, hydroxypyridinone, competition, luminescence spectroscopy, nuclear magnetic resonance spectroscopy, thermodynamic modelling

## Abstract

Incorporation of lanthanide (Ln) and actinide (An) ions into the human body poses significant chemotoxic and radiotoxic risks, necessitating effective decorporation strategies. This study investigates the displacement of biologically relevant ligands from trivalent ions of europium, Eu(III), and curium, Cm(III), in artificial biofluids by various complexing agents, i.e., ethylenediaminetetraacetic acid (EDTA), ethylene glycol-bis(*β*-aminoethyl ether)-*N*,*N*,*N*′,*N*′-tetraacetic acid (EGTA), diethylenetriaminepentaacetic acid (DTPA), and spermine-based hydroxypyridonate chelator 3,4,3-LI(1,2-HOPO) (HOPO). Utilizing a modified unified bioaccessibility method (UBM) to simulate gastrointestinal conditions, we conducted concentration-dependent displacement experiments at both room and body temperatures. Time-resolved laser-induced fluorescence spectroscopy (TRLFS) supported by ^2^H nuclear magnetic resonance (NMR) spectroscopy and thermodynamic modelling revealed the complexation efficacy of the agents under physiological conditions. Results demonstrate that high affinity, governed by complex stability constants and ligand p*K*_a_ values, is critical to overcome cation and anion competition and leads to effective decorporation. Additionally, there is evidence that cyclic ligands are inferior to linear ligands for this application. HOPO and DTPA exhibited superior displacement efficacy, particularly in the complete gastrointestinal tract simulation. This study highlights the utility of in vitro workflows for evaluating decorporation agents and emphasizes the need for ligands with optimal binding characteristics for enhanced chelation therapies.

## 1. Introduction

Lanthanide (Ln) and actinide (An) ions can enter the environment in various ways. Ln are detectable in rivers and ground waters of their mining areas [1,2,3], while An or other radionuclides (RNs) are released into the biosphere through mining activities, the widespread use of phosphate fertilizers, medical applications, and incidents at nuclear power or processing facilities as well as repositories [4,5,6,7,8,9,10,11,12,13]. These metals can enter the human body through inhalation, absorption, or ingestion, proven by detection of Ln in the blood and hair of the inhabitants of mining regions [14,15]. Once incorporated, they pose serious health risks. Both Ln and An exhibit chemotoxic effects, while An additionally cause radiotoxic damage of bones, liver, lung, and kidneys [16,17]. After accumulating in a target organ, these metals can damage the surrounding cells, potentially inducing necrosis or carcinogenesis [18,19,20,21]. In addition, physiologically active cations such as Ca^2+^ can be displaced by Ln(III) and An(III), leading to a reduced activity of certain enzymes [22,23]. To mitigate these risks, it is necessary to promote the excretion of the metals. This can be achieved by chelation therapy, a medical procedure that provides binding partners that compete with the endogenous biological ligands and convert the metal ions into easily excretable forms. Such binding partners are typically multidentate organic compounds commonly referred to as chelating or decorporation agents (DAs). It has been traditionally used to treat heavy metal poisoning (e.g., lead, cadmium, mercury, arsenic) [24,25,26,27,28,29], and in some cases, iron overload in conditions like thalassemia [30,31]. Different DAs are employed depending on the nature of the metal. According to Pearson’s Hard and Soft Acids and Bases (HSAB) principle [32], DAs with soft donor atoms like dimercaptopropane sulfonate (DMPS) or dimercaptosuccinic acid (DMSA) are effective against soft metals such as arsenic or mercury [24,25,26], whereas DAs with hard binding donor atoms like ethylenediaminetetraacetic acid (EDTA) and diethylenetriaminepentaacetic acid (DTPA) are preferred for hard metal ions like lead or Ln(III)/An(III) [27,28,29]. EDTA and DTPA have been tested or are in use as decorporation agents against plutonium, americium, and curium incorporation [28,33,34,35,36,37,38,39,40,41,42,43]. However, various studies reported certain drawbacks associated with these ligands, such as intrinsic toxicity, low absorption in the gastrointestinal tract, and poor complexation behaviour towards higher valent actinides such as Pu(IV), Np(V), and U(VI) [44]. Recent developments focus on enhancing selectivity, bioavailability, and patient safety, particularly in the fields of nuclear medicine, environmental exposure, and emergency preparedness. New formulations of established chelators such as DTPA are being designed to improve oral bioavailability [42,45,46], while novel ligands with high specificity—especially for actinides—are being developed to enable more targeted and effective decorporation [28,38]. A promising new DA, which is currently in clinical trials, is the spermine-based hydroxypyridonate octadentate chelator 3,4,3-LI(1,2-HOPO), in the following abbreviated as HOPO. It is orally active, less toxic than agents such as DTPA, and exhibits high selectivity, particularly for multivalent radionuclides, especially actinides [47,48,49,50].

For the further development of decorporation strategies, understanding the competition between DAs and endogenous biological ligands at the molecular level is of fundamental importance. Focusing on oral administration, in this study the interaction of a series of model DAs with metal ions was investigated in artificial biofluids of the digestive system based on the unified bioaccessibility method (UBM) developed by the Bioaccessibility Research Group of Europe (BARGE) [51] and modified by Wilke et al. [52]. This protocol simulates the gastrointestinal tract using four artificial fluids—saliva, gastric juice, pancreatic juice, and bile fluid—combined in defined ratios. The resulting mixture reflects conditions in the small intestine, where nutrient and heavy metal uptake occurs [16,17,53,54]. To prevent absorption of these chemo- and radiotoxic elements, they must be converted into species favoring excretion over uptake.

**Figure 1:**
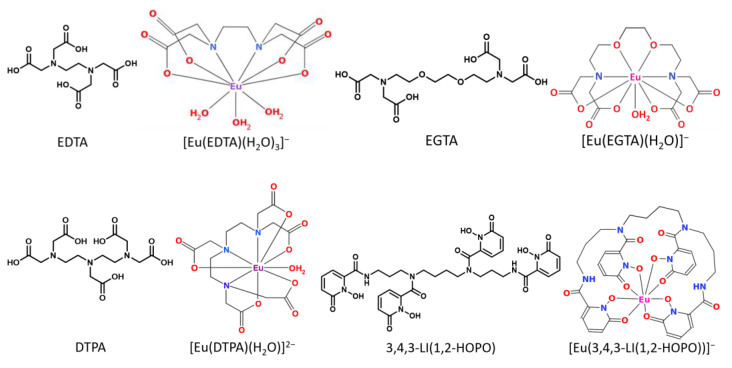
Generic structures of the potential decorporation agents and their Eu(III) complexes considered in this study.

Among Ln(III) and An(III), europium, Eu(III), and curium, Cm(III), have been selected due to their redox stabilities and chemical properties. With ionic radii of approximately 109 and 99 pm [55], respectively, they are typical representatives of Ln(III) and An(III). Both exhibit excellent luminescence properties enabling time-resolved laser-induced fluorescence spectroscopy (TRLFS) at environmentally relevant concentrations [20]. The DAs chosen for this work are the aminocarboxylates EDTA, ethylene glycol-bis(β-aminoethyl ether)-*N*,*N*,*N′*,*N′*-tetraacetic acid (EGTA), and DTPA, which selectively coordinate trivalent cations, as well as the hydroxypyridonate HOPO, which offers improved oral bioavailability, lower toxicity, and enhanced selectivity for tri- to hexavalent actinides [47,48,49,50]. Their general structures, as well as their 1:1 complexes with Eu(III) and the corresponding coordination sites, are shown in Figure 1. The 1:1 complexes differ in denticity (number of coordination sites) and the nature of binding sites (type of functional groups) [56,57,58,59,60]. The proposed complex structures in solution are similar to comparable crystal structures [61,62,63,64,65,66].

Naturally occurring Ca(II) in digestive biofluids behave similarly to Ln(III) and An(III), competing for DA binding sites. Conversely, anionic ligands, especially phosphate, are favorable binding partners for Eu(III) and Cm(III) and have to be displaced by the DA. Therefore, both cation and anion competition must be overcome to achieve effective and selective DA–RN complexation.

## 2. Results and Discussion

The composition of the artificial digestive system was adapted from Wilke et al. (see Table 1) [51,52].

**Figure 2:**
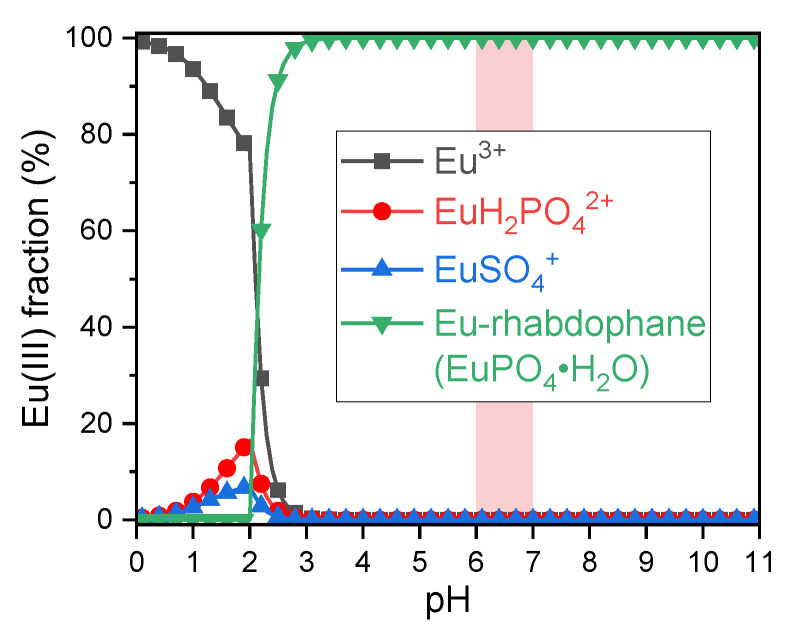
Thermodynamic modelling of the Eu(III) speciation in the whole artificial digestive mixture. Only inorganics were taken into account due to a lack of thermodynamic data for organic biological ligands. Marked in red is the pH that occurs naturally in the small intestine.

Recent studies have indicated that the primary binding partners for trivalent europium and curium are phosphate, as well as the proteins mucin and amylase [22,52,67]. Thermodynamic modelling of the inorganic fraction also indicates that phosphate plays a pivotal role (see Figure 2). Due to lack of thermodynamic data, organics and enzymes are omitted.

In light of the available literature data and the results of the thermodynamic modelling, two approaches were pursued. In the initial approach, only the phosphate fraction of the GIT was selected. In the second experimental series, the solution combined all GIT components. To further validate the reliability of the workflow, each series was conducted at both room temperature and body temperature.

To determine the efficacy of the DA in quantitatively displacing the biological ligands, concentration-dependent experiments were performed in the artificial digestive system and monitored by TRLFS. The fraction of Eu bound to the corresponding DA was determined. To extend the results on An(III), in a different approach, Eu(III) was replaced by Cm(III) and the samples were also investigated using TRLFS. Complementary qualitative experiments using ^2^H-NMR spectroscopy were performed for additional insight into the displacement of biological ligands from the metal’s coordination sphere from the chelating ligand’s perspective.

### 2.1. Kinetics of the Displacement Reactions

**Figure 3:**
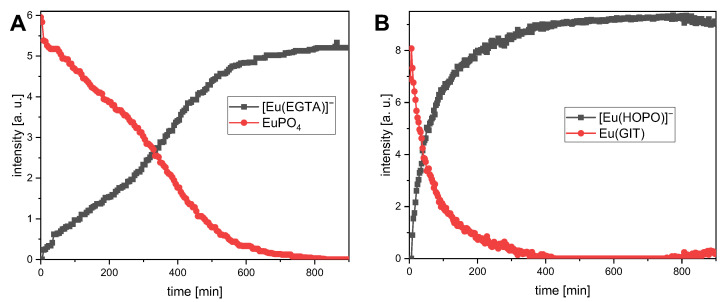
Time-dependent displacement of phosphate by EGTA (**A**) and the complete GIT mixture by HOPO (**B**) from the coordination sphere of Eu(III) measured by TRLFS. (**A**): [Eu(III)] = 10 µM, [EGTA] = 15 mM, [phosphate] = 14.8 mM, pH = 6.5 ± 0.5, *T* = 37 °C. (**B**): [Eu(III)] = 10 µM, [HOPO] = 1 mM, complete GIT mixture (see Table 1), pH = 6.5 ± 0.5, *T* = 37 °C.

The kinetics of the biological ligands displacement by selected DAs was investigated first. For doing so, TRLF spectra were measured at regular intervals over a period of 15 h. Two approaches were pursued: in the first, only phosphate at its highest concentration typically found in the body fluids was mixed with Eu(III) and EGTA. In a second attempt, the complete GIT was mixed with Eu(III) and HOPO. The obtained spectra were analyzed applying parallel factor analysis (PARAFAC, for more information see Section 3), which yielded the time-dependent displacement of any bioligands from the GIT by the DAs, as shown in Figure 3.

Two major results are evident from the curves. First, both ligands are able to quantitatively displace the respective bioligands from the metal ion. Second, it requires at least 5 h (Figure 3B) up to 11 h (Figure 3A) to fully displace the bioligands. This corresponds to the time span of up to 10 h required for food to pass through the small intestine [68]. Based on these findings, an overnight equilibration step was implemented in all subsequent experiments to account for kinetic limitations.

### 2.2. Displacement of the Phosphate Fraction of the GIT

**Figure 4:**
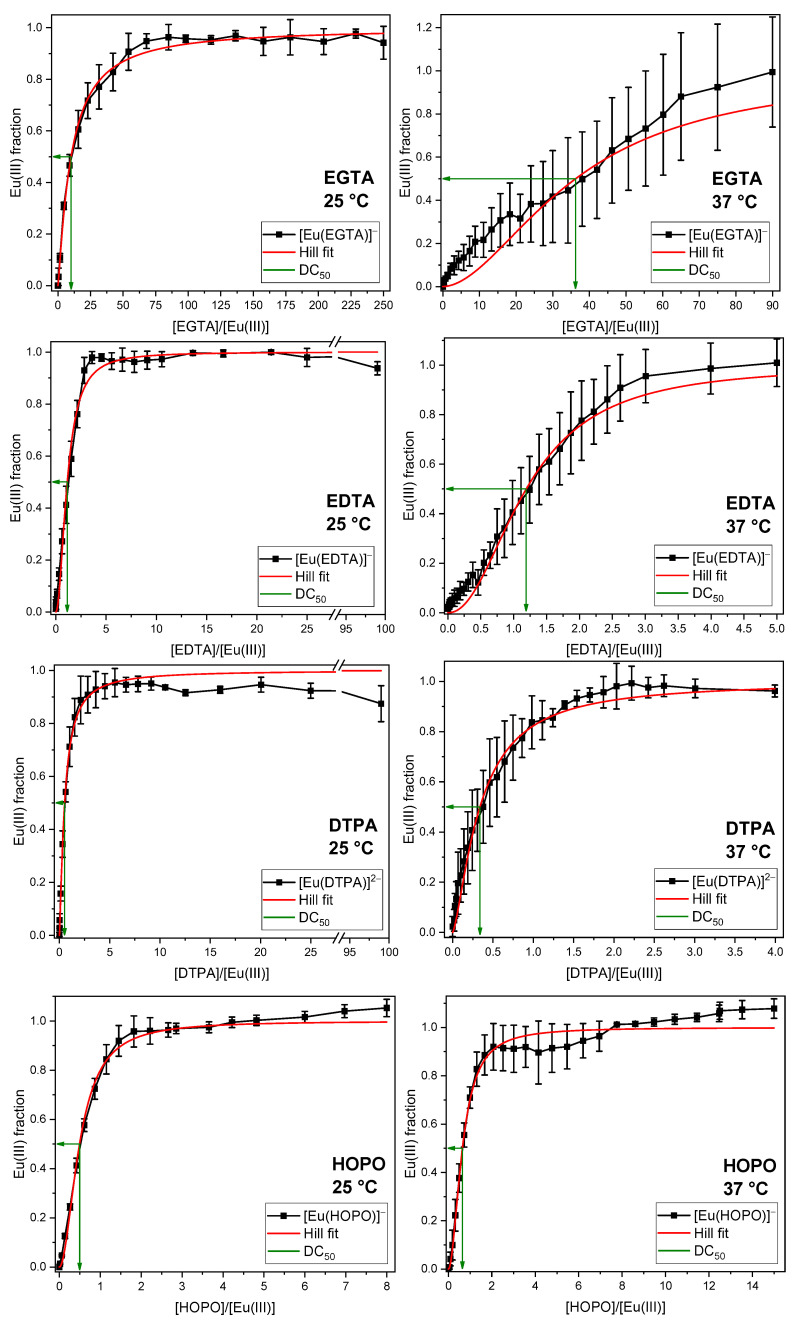
Displacement of the phosphate fraction of the artificial digestive system as a function of the EGTA, EDTA, DTPA, and HOPO concentrations at room temperature (**left**) and body temperature (**right**). [Eu(III)] = 10 µM, [phosphate] = 3.73 mM, *I* (NaCl) = 344 mM, pH = 6.5 ± 0.5.

The phosphate concentration occurring in the GIT was mixed with Eu(III) and varying DA concentrations. After overnight equilibration, TRLF spectra were measured and analyzed by PARAFAC. The obtained fractions of the Eu(III)–DA complex as a function of the DA/Eu(III) ratio are given in Figure 4. Each DA was investigated at room (25 °C) and body temperature (37 °C). Further details on experimental results are given in the Supplementary Materials, Figures S2–S9 and Tables S1–S8.

To quantify the efficacy of the different DAs and to facilitate comparison, the excess of DA at which half of the Eu(III) or Cm(III) was bound to the complex was calculated (decorporation concentration, DC_50_). For additional information, see Section 3.

In each series, the Eu(III) complex formed with the corresponding DA reaches 100% of the europium fraction, indicating that all DAs are capable of completely displacing the phosphate from Eu(III) at room and body temperatures. In some series, the fractions of Eu(III) species exceed 100%. This is attributed to the significant disparity in the quantum yield, luminescence intensity of the europium phosphate and the Eu(III)–DA complexes, and the underlying model used for the PARAFAC analysis. The luminescence lifetimes and emission spectra of the corresponding complexes are in good agreement with the reference values (see Table 2 for lifetimes and Supplementary Figures S2, S4, S6 and S8 for emission spectra). The primary distinction between the various ligands lies in their concentrations required to displace phosphate. The DC_50_ values of the four ligands are presented in Table 3. They show almost no temperature dependence, except for EGTA, where the DC_50_ value increases with temperature, which appears counterintuitive. Among the DAs investigated, EGTA is a rather weak complexing agent owing to its comparatively high (sum of) p*K*_a_ values. As a result, small variations in pH caused, for example, by phosphate buffering under the chosen conditions or by the fact that each sample was prepared individually, can lead to small but significant differences in [Eu(EGTA)]^−^ complex quantities forming.

To validate the experimental findings, the DC_50_ values were thermodynamically modelled (see Figures S20–S25 and Tables S17–S22 Supplementary Materials). The experimentally determined DC_50_ values reflect the same order as the thermodynamically modelled ones, with the highest DC_50_ value for EGTA and the lowest for DTPA and HOPO. The thermodynamic calculations show a DC_50_ value of EDTA 10-fold lower than of EGTA, as was inferred from the experimental results. For DTPA and HOPO, the modelled and observed DC_50_ values match strikingly, which is due to their high affinity for Eu(III).

### 2.3. Displacement of All Components of the GIT

In order to simulate the digestive system as accurately as possible, in the following experiments the DA were used against all components of the GIT (see Table 1). Again, each ligand was analyzed at both 25 and 37 °C (Figure 5; for further experimental details see Figures S10–S17 and Tables S9–S16, Supplementary Materials).

In accordance with the findings on phosphate displacement described above, it can be concluded that all DAs are capable of displacing the bioligands present in the GIT from the coordination sphere of europium at both room and body temperature. Moreover, an even more extensive dispersion of the data is evident. This is attributable to the nature of the solutions, which were not clear, but instead exhibited turbidity (see Figure S34, Supplementary Materials). The obtained lifetime of the Eu(III) species in GIT was determined to be 315 ± 52 µs, which is in good agreement with the literature data of europium complexes with various enzymes (Table 2), and supports the conclusion that Eu(III) in the GIT fraction is predominantly bound to enzyme proteins. Lifetimes of the Eu(III) complexes of the several DAs are also in agreement with the literature values (see Table 2).

The measured DC_50_ values of the four DAs are presented in Table 3. Interestingly, all DAs show increased DC_50_ values in the whole GIT compared to the phosphate fraction. It seems that the enzymes bind the europium in addition to phosphate, which leads to an increased amount of complexing agent required to release the metal. This was reported earlier by Wilke et al. [52] and is also evident in the emission spectra: the spectra of Eu(III) in the GIT fraction without any DA differ significantly from those of Eu(III) in the DA-free phosphate fraction, despite identical phosphate concentrations in both cases (Figures S10, S12, S14, and S16, Supplementary Materials for emission spectra). Additionally, a temperature dependence is observable. At body temperature, all DC_50_ values are (much) smaller than at room temperature. This reflects the increased exchange dynamics in the system due to the increased temperature according to the Van’t Hoff equation.

To further expand the existing knowledge on trivalent actinides, Cm(III) was investigated using a similar experimental approach (see Figure 6). Due to the need for strict safety measures and limited resources, each experimental series was performed only once under conditions designed to be as realistic as possible (full artificial GIT, body temperature).

**Figure 5:**
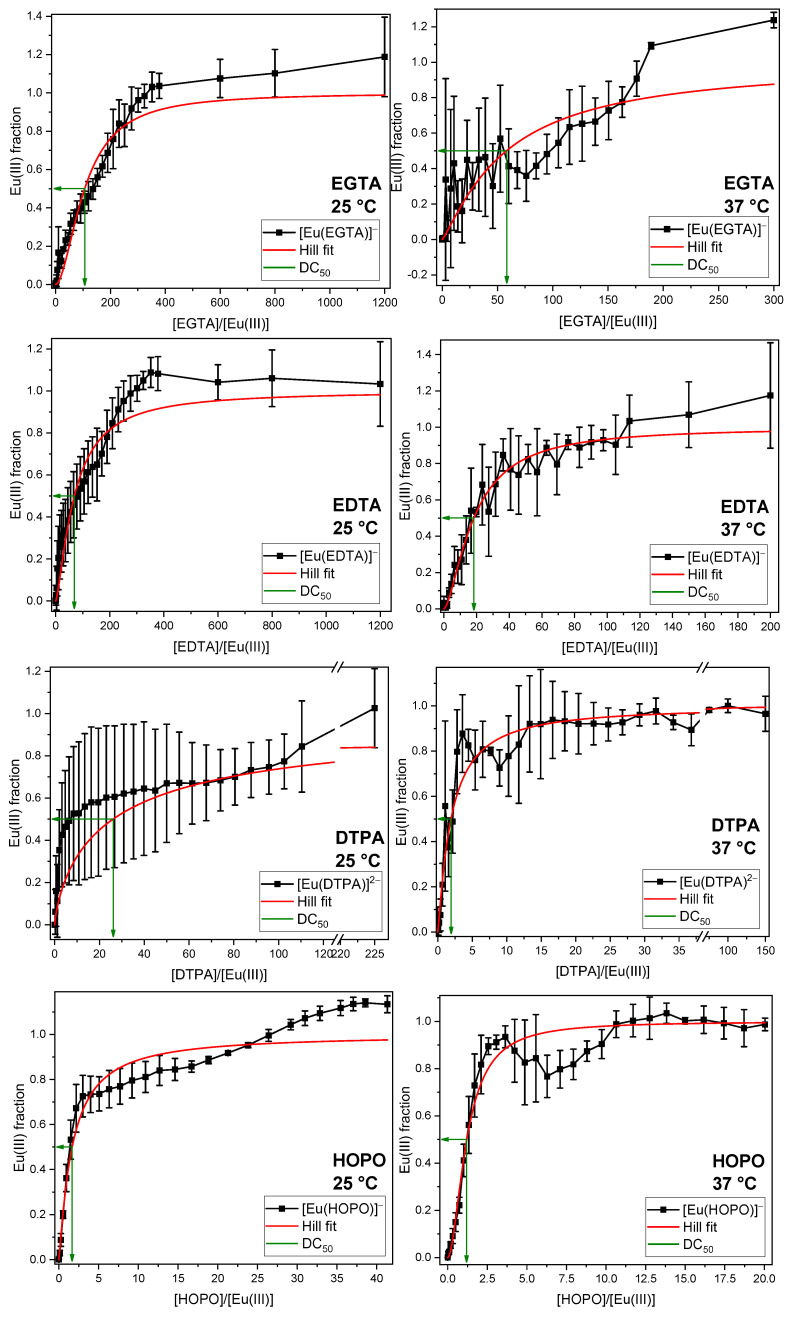
Displacement of all components of the artificial GIT as a function of the EGTA, EDTA, DTPA, and HOPO concentrations at room temperature (**left**) and body temperature (**right**). [Eu(III)] = 10 µM, pH = 6.5 ± 0.5.

**Figure 6:**
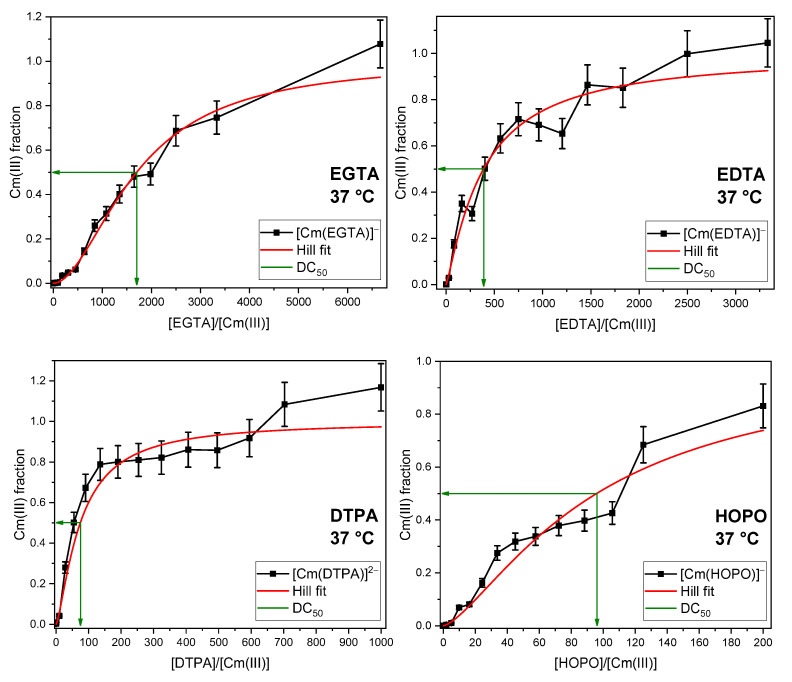
Displacement of all components of the artificial GIT as a function of the DA concentration at body temperature. DAs are EGTA, EDTA, DTPA, and HOPO; [Cm(III)] = 0.3 µM, pH = 6.5 ± 0.5.

The emission spectra of the species formed between Cm(III) and the binding partners from the GIT (Figure S19, Supplementary Materials) appear broad and comparably noisy, even after extraction from the sum spectra by PARAFAC. There are various reasons for this. First, Cm(III) is bound to multiple binding partners according to Wilke et al. [52], which are primarily the enzymes amylase and mucin. Each of these species leads to one emission peak. Since multiple species coexist, superposition of all peaks results in a broad feature. Second, as already observed in the example of Eu(III), the GIT mixture scatters and absorbs the emitted light, resulting in noisy spectra. This is typical for this kind of experiment, and has already been reported in previous studies [22,52,67].

Similar to the Eu(III) results, all DAs achieved complete complexation of the Cm(III) in the GIT mixture, albeit with substantial differences in their DC_50_ values (cf. Table 3).The luminescence lifetimes, as well as the spectral features of the Cm(III) complexes with bioligands from the GIT, EGTA, EDTA, DTPA, and HOPO, agree well with the literature data for Cm(III) with biological binding partners and chelating agents (see Table 2). A pronounced difference is observed in the excess of DA required to displace the biological ligands compared to the respective Eu(III) experiments. This can be attributed to the significantly lower Cm(III) concentrations, which further reduce the effective affinity of the ligands.

### 2.4. Thermodynamic Correlation of the Displacement Efficacy

In order to analyze the relationship between the efficacy of a DA and its thermodynamic and complex formation properties, a series of relevant parameters was collected for each ligand. The p*K*_a_ values and denticity of the ligands and complex formation constants (log *K*_ML_) for the fully deprotonated 1:1 complexes with Eu(III) and Cm(III), as well as Ca(II), are presented in Table 4.

**Figure 7:**
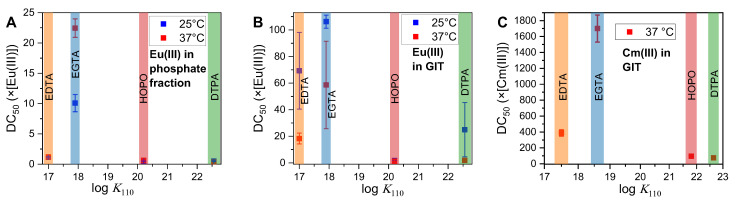
DC_50_ values of each ligand obtained by displacing experiments in comparison to the complex stability constants log *K*_110_ (corresponding to the stoichiometric coefficients of metal ion, ligand, and protons, respectively; see Table 4) of the corresponding complex. (**A**): Eu(III) in phosphate fraction, (**B**): Eu(III) in complete GIT, (**C**): Cm(III) in complete GIT. *T* = 25 °C (blue) or 37 °C (red).

In almost all series, the DC_50_ values of the four DAs are in the order EGTA >> EDTA > DTPA ≈ HOPO. This finding is not consistent with the reverse order of the corresponding complex stability constants, which indicates that EDTA forms the least stable complexes, followed by EGTA, HOPO, and DTPA (see Figure 7).

These findings indicate that the log *K* value is not the sole determinant of the displacement efficacy of each ligand. It is crucial to take into account the pH and (the sum of) p*K*_a_ values of each ligand. Given that the metal cation (Eu(III) or Cm(III)) competes with the protons at the coordination sites of the ligand, it is evident that the concentration of protons (=pH) cannot be disregarded. As indicated in the literature, a pH-dependent (“conditional”) dissociation constant (*K*_d,M_) can be calculated from the complex stability constants (log *K*_110_ = log *K*_M_) and the p*K*_a_ values of the ligand [73]:

(1) $$K_{d,M}=\frac{1+10^{{pK}_{a1}-pH}+10^{{pK}_{a2}+{pK}_{a1}-2\cdot pH}}{K_{M}}$$

Since Equation (1) only takes into account the first two p*K*_a_ values, it can be generalized for any ligand with *n* p*K*_a_ values:

(2) $$K_{d,M}=\frac{1+\sum_{k=1}^{n}10^{\sum_{i=1}^{k}{pKa}_{i}-k\cdot pH}}{K_{M}}$$

The reciprocal of *K*_d,M_ is the conditional affinity constant (*K*_a,M_):

(3) Ka,M=1Kd,M=KM1+∑k=1n10∑i=1kpKai−k·pH

**Figure 8:**
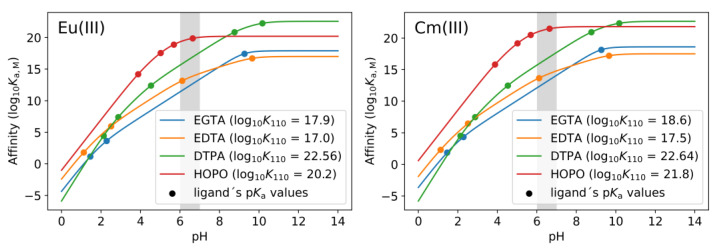
Visualization of Equation (3) for the four DAs used in this study. **Left**: affinities for Eu(III); **right**: affinities for Cm(III). Grey area marks the pH occurring in the artificial GIT (6.5 ± 0.5).

**Figure 9:**
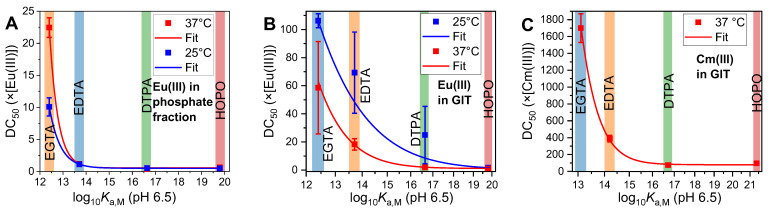
DC_50_ values of each DA obtained by displacing experiments in comparison to the conditional affinity constants of the corresponding complex. (**A**): Eu(III) in phosphate fraction, (**B**): Eu(III) in complete GIT, (**C**): Cm(III) in complete GIT. *T* = 25 °C (blue) or 37 °C (red).

Applying Equation (3) to the parameters of the Eu(III) and Cm(III) complexes of the four DAs in this study (Table 4) shows the pH dependence of the affinity (see Figure 8):

The calculated affinities of each ligand for the two metals are presented in Table 5. A more straightforward trend emerges when these values are correlated with the DC_50_ values obtained (Figure 9). A higher affinity for Eu(III) or Cm(III) results in a lower concentration of DA required to displace any GIT-derived bioligand, such as phosphate or proteins. If the affinity is sufficiently high, as is the case with DTPA and HOPO, an upper limit is reached, whereby the addition of almost every molecule of ligand results in the capture of a metal cation. Subsequently, the concentration of DA necessary to attain the DC_50_ is found to be half the concentration of the metal (DC_50_ = 0.5 × [Eu/Cm(III)]). This precise phenomenon was observed exclusively in the context of Eu(III) within the phosphate fraction.

The correlation between the DC_50_ values and conditional affinities were fitted using an asymptotic equation (Equation (4)). The resulting parameters for all fits are given in Table 6.

(4) $${DC}_{50}\left(\times [M(III)]\right)=A+B\cdot C^{{log}_{10}K_{a,M}}$$

Parameter B in Table 6, corresponding to Equation (4), shows large errors, which can be attributed to several factors. First, the scale of the fits in Figure 9 is logarithmic and the endpoint is unknown. This means that for ligands with relatively low affinity towards the metals, an exponentially increasing concentration is required to displace the bioligands from the metal’s coordination sphere, which is not applicable for medicinal purposes. Second, since the sample solutions were turbid (see Figure S34, Supplementary Materials) and contain a high concentration of enzymes, the observed uncertainty and scattering in the data are to be expected in biological environments, as shown in the case of U(VI) with milk proteins [74]. For Cm(III), much higher ligand excess is required to displace the bioligands from its coordination sphere. This is due to the significantly lower concentration of curium in comparison to europium and the definition of the *K*_D_ [75].

With these parameters and the affinity of any comparable ligand, its decorporation efficacy can be estimated for any ligand with known complex stability constant and p*K*_a_ values.

### 2.5. Applicability to Other Complexing Agents

**Figure 10:**
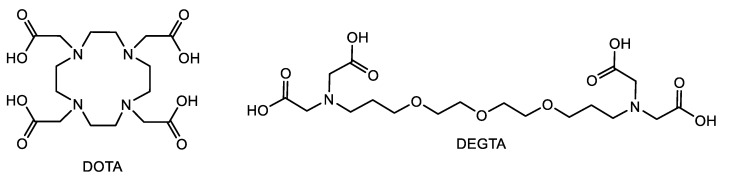
General structure of the ligands DOTA and DEGTA.

Additional DAs, which fit in this series, are, for example, 2,2′,2″,2‴-(1,4,7,10-Tetraazacyclododecane-1,4,7,10-tetrayl)tetraacetic acid (DOTA) [76,77] and diethylene glycol-bis(3-aminopropyl ether)-*N*,*N*,*N′*,*N′*-tetraacetic acid (DEGTA) [78] (see Figure 10). DOTA is already widely used in radiopharmaceutical application for cancer treatment and diagnosis (as complex with ^90^Y) [79,80] and as contrast agent (as Gd complex) [81,82]. Additional radiolabeled complexes are described, for example, using ^44^Sc [83], ^68^Ga [84], ^111^In, and ^177^Lu [85]. This and its commercial availability make it a suitable compound for this purpose. DEGTA represents an extension of EDTA and EGTA with similar binding motifs [78], which makes it an interesting candidate for this investigation.

Although DOTA has a high affinity for europium (log *K*_a,M_ = 16.3 calculated using Equation (2) with data from refs. [86,87]), from which a DC_50_ value of 10 × [Eu(III)] at room temperature is calculated using Equation (4), even much higher concentrations (up to 50 × [Eu(III)]) are not sufficient to displace europium from the GIT constituents (see Figure S18, Supplementary Materials). DOTA appears to bind other cations first, especially Ca(II), which is present in a 200-fold excess compared to Eu(III) (see Table 1). At this point, another cation cannot be complexed by the ligand because the exchange reaction is kinetically hindered. Therefore, it seems that these results can only be extrapolated to comparable acyclic ligands.

DEGTA, on the other hand, has a low affinity at the pH values given in the GIT mixture (log *K*_a,M_ = 9.7, calculated using Equation (3) with data from Friedrich et al. [78]), which is attributable to the steric hindrance of the extended backbone even if the binding occurs with all nine possible binding sites. The affinity corresponds to a calculated DC_50_ of 551 × [Eu(III)], which is not suitable for decorporation purposes. This high value is based on calculations according to Equation (4). Recently published experiments of the fast displacement of DEGTA from the coordination sphere of Eu(III) by EGTA at room temperature supports this thesis [78].

These examples show the suitability, but also limitations, of the proposed estimation. However, it can be a helpful tool to reasonably plan experiments and minimize experimental efforts.

### 2.6. Investigations from the Ligand’s Perspective

**Figure 11:**
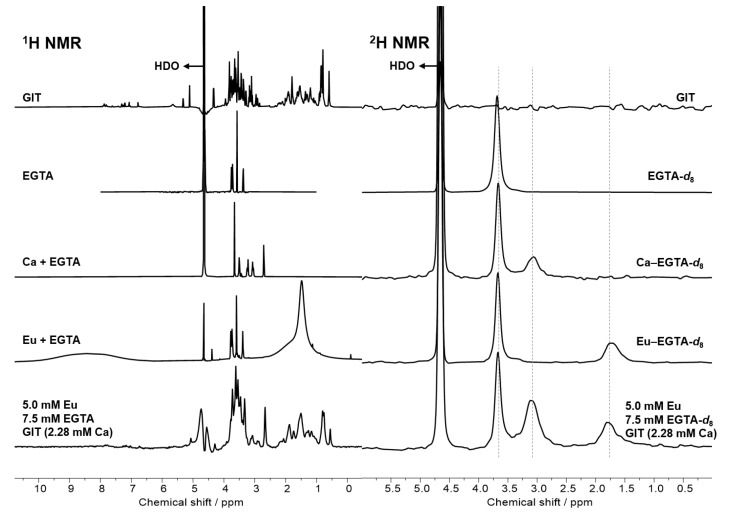
^1^H (**left**) and ^2^H NMR (**right**) spectra of free EGTA, Ca(II)-, and Eu(III)-bound EGTA and GIT with and without Eu or EGTA.

So far, the investigation has aimed at the coordination sphere of the metal ions. In order to verify the TRLFS results by a complementary method and to have a view from the ligand’s perspective, NMR spectroscopy was employed. The presence of organics and, in particular, of proteins in the total mixture (GIT) results in a significant and challenging ^1^H NMR signal background, which makes the observation of any DA difficult. The numerous (macromolecular) organic molecules of the GIT mixture give rise to manifold ^1^H signals hampering detection of EGTA’s signals, regardless of speciation (Figure 11, left). From these spectra, no qualitative statement is possible, let alone a quantitative one. Recently published methods for straightforward access to deuterated ligands address this issue to apply ^2^H NMR spectroscopy [88]. Due to the low natural abundance of deuterium, the impact of organics and proteins is negligible. Given the considerably higher concentrations typically required for NMR spectroscopy, these experiments were not conducted under environmental conditions. However, they are designed to reinforce the general trend observed above. The deuterated form of EGTA, EGTA-*d*_8_, was selected for this series as a proof-of-concept example.

**Figure 12:**
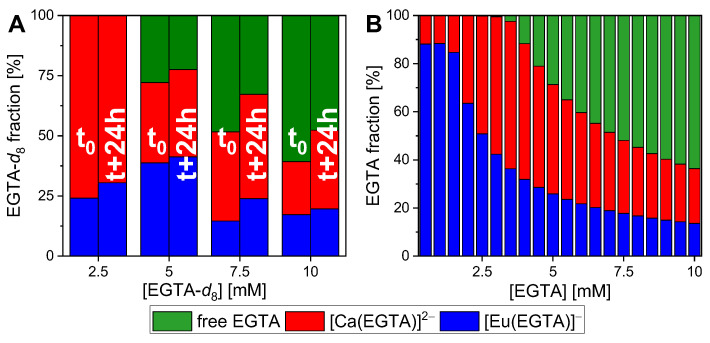
Fractions of free, Ca(II)-, and Eu(III)-bound EGTA in the artificial GIT mixture measured by ^2^H-NMR spectroscopy (**A**) and modelled using PHREEQC (**B**). Each sample was measured immediately after addition of the ligand and the next day. [Ca(II)] = 2.28 mM, [Eu(III)] = 5 mM, pH = 6.5 ± 0.5.

As illustrated in the right side of Figure 11, the ^2^H NMR spectra of EGTA-*d*_8_, which exhibit a single signal per species, can be readily deconvoluted into free, Ca(II)-, and Eu(III)-bound EGTA. This allows the quantity of EGTA required to release the europium from the bioligands to be determined. To simulate the duration of a digestion process, the samples were measured twice: once immediately after the addition of the ligand and again the following day. As in the TRLFS experiments, the ligand concentration was varied (see Figures S26–S33, Supplementary Materials). The fractions obtained for each ligand concentration at both time points, as well as values obtained from thermodynamic modelling, are presented in Figure 12 (for values see Table S23, Supplementary Materials).

The results demonstrate the cation competition between the Ca(II) and Eu(III) ions for ligand binding, mirroring their similar chemical behaviour mainly caused by almost identical ionic radii. Moreover, as demonstrated by the time-dependent data (see Figure 3), the fractions of both metal–DA complexes increased over the course of a day. The metal ions are bound by a variety of bioligands, including phosphate, carbonate, and enzymes such as amylase. Their displacement and subsequent complexation by EGTA demonstrate a time-dependent behaviour. Even high EGTA concentrations (twice the Eu(III) concentration) are unable to bind all Eu(III). This was also demonstrated by both TRLFS measurements, where a much higher EGTA excess (>100 times the Eu(III) concentration) was employed, and thermodynamic modelling as shown in Figure 12B.

## 3. Materials and Methods

### 3.1. Starting Material and Stock Solutions

Caution! Curium is a highly radioactive element requiring special precautions for handling, and all studies were conducted in a laboratory dedicated to actinide research.

HOPO was synthesized as previously reported with slight modification. The details are presented in the Supplementary Materials (Synthesis: Scheme S1; ESI-MS, ^1^H NMR and FT-IR characterization: Figure S1) [41,89,90,91,92]. The other chemicals were used as obtained. Stock solutions were prepared by weighing and dissolving appropriate amounts of EuCl_3_∙6H_2_O (99.99%, Sigma-Aldrich, Taufkirchen, Germany), H_4_EDTA (>99%, Roth, Karlsruhe, Germany), H_4_EGTA (≥99%, Roth, Karlsruhe, Germany), H_5_DTPA (>99%, Fluka-Feinchemikalien GmbH, Neu-Ulm, Germany), NaCl (99.5%, Roth, Karlsruhe, Germany), KCl (p.a., Merck, Darmstadt, Germany), NH_4_Cl (99.5%, Sigma-Aldrich, Taufkirchen, Germany), MgCl_2_∙6H_2_O (>99%, Roth, Karlsruhe, Germany), CaCl_2_∙2H_2_O (99%, Roth, Karlsruhe, Germany), NaH_2_PO_4_∙H_2_O (anhydrous, Merck, Darmstadt, Germany), KH_2_PO_4_ (≥99%, Roth, Karlsruhe, Germany), NaHCO_3_ (≥99%, Roth, Karlsruhe, Germany), KHCO_3_ (p.a., Merck, Darmstadt, Germany), Na_2_SO_4_ (≥99%, Roth, Karlsruhe, Germany), KSCN (p.a., Riedel-de Haen, Seelze, Germany), urea (99.5%, Acros, Geel, Belgium), uric acid (99%, Acros, Geel, Belgium), glucose (p.a., Roth, Karlsruhe, Germany), d-(+)-glucosamine hydrochloride (99.5%, Merck, Darmstadt, Germany), and glucuronic acid (99.5%, Thermo Fisher, Dreieich, Germany) in Milli-Q H_2_O (18.2 MΩ cm, Millipore, Merck, Darmstadt, Germany). The enzymes mucin (75–95%, Roth, Karlsruhe, Germany), pepsin (from porcine gastric mucosa, Thermo Fisher, Dreieich, Germany), α-amylase (from porcine pancreas), pancreatin (from porcine pancreas), trypsin (from bovine pancreas), lipase (from Rhizopus oryzae), and bile extract (bovine, all Sigma-Aldrich, Taufkirchen, Germany) were weighted and added as obtained. pH was adjusted with HCl (1.0, 0.1, and 0.01 M) and NaOH (1.0, 0.1, and 0.01 M), and in D_2_O solutions likewise DCl and NaOD (both >99% D, Deutero, Kastellaun, Germany), using a pH meter (inoLab pH 730, Xylem, Weilheim, Germany) equipped with a pH electrode (SCHOTT, BlueLine, SI Analytics, Mainz, Germany).

^248^Cm was obtained from the transplutonium element production facilities at Oak Ridge National Laboratory, Oak Ridge, TN, USA. Appropriate dilutions were made from a 295 μM Cm(ClO_4_)_3_ stock solution.

### 3.2. Preparation of the Artificial Digestive System

The composition of the artificial digestive system was adapted from Wragg et al. and Wilke et al. [51,52]. Instead of adding the single biofluids step by step, the components from the final GIT mixture were directly combined, either only the phosphate fraction or all inorganics, organics, and enzymes. Afterwards, Eu(III) or Cm(III) was added, pH adjusted to 6.5 ± 0.5 using HCl or NaOH, and the solution was vigorously shaken for about one hour. To evaluate the ability of different DAs to displace bioligands from the coordination sphere of Eu(III) or Cm(III), the concentration of DA was gradually increased while the concentrations of the bioligands (either the phosphate alone or the full GIT mixture), of Eu(III) or Cm(III), and the pH were kept constant. When examining the phosphate fraction alone, the ionic strength was taken into account. For doing so, aliquots of the metal-containing GIT solution were taken and the corresponding amount of DA was added. To simulate the digestion process, which takes multiple hours, the samples were shaken overnight at a constant temperature. The following day, the pH of the samples was checked (and adjusted, if necessary) and the samples were measured using TRLFS at constant temperature. For the ^2^H NMR experiments, the samples were prepared in the same manner but measured twice: directly after preparation as well as on the day after.

### 3.3. Quantification of the Displacement Efficacy

The fraction of the Eu(III)- or Cm(III)–ligand complex obtained from the speciation was correlated with the excess of DA. The curves were fitted using a Hill fit, and for a more accurate fit, the upper limit was set at 100% Eu(III)/Cm(III)–DA complex, which corresponds to a fraction of 1 (Equation (5)).

(5) $$Eu(III)/Cm\left(III\right)fraction=\frac{({\left[DA\right]/[M\left(III\right)])}^{n}}{{k^{n}\cdot ([DA]/[M\left(III\right)])}^{n}}$$

Equation (5) was rearranged according to [DA]/[M(III)] with an Eu/Cm(III) fraction of 0.5, equaling to 50%.

(6) $${DC}_{50}={\left(\frac{0.5\cdot k^{n}}{1-0.5}\right)}^{\frac{1}{n}}={\left(k^{n}\right)}^{\frac{1}{n}}$$

### 3.4. NMR Spectroscopy

NMR spectra were obtained at (25 ± 0.2) °C with Agilent DD2-600 and MR-400 systems (Agilent Technologies, Waldbronn, Germany), operating at 14.1 as well as 9.4 T, with corresponding ^1^H and ^2^H resonance frequencies of 599.8 and 92.1 MHz, as well as 399.8 and 61.4 MHz, respectively, using 5 mm oneNMR probes. Chemical shifts are reported in parts per million relative to external TMS for ^1^H. ^2^H spectra are referenced to the HDO chemical shift observed in the corresponding ^1^H NMR spectrum. Data acquisition was performed according to the procedure described in Friedrich et al., 2025 [88]. Samples containing the GIT components were prepared according to the composition listed in Table 1. To these solutions, EGTA-*d*_8_ was added corresponding to its target concentration. EGTA-*d*_8_ stock solution was prepared from weighing crystalline material in H_2_O obtained from Lu(III)-catalyzed selective deuteration of the aminoacetate methylene carbons, described elsewhere [88]. Eu(III) was finally added, and the samples measured twice. The first measurement was immediately after preparation. The second measurement was after 24 h with samples kept at 37 °C in a water bath.

### 3.5. Luminescence Spectroscopy

Solutions were stirred in 10 mm path length Hellma Analytics 4 mL quartz cells. The cuvette was placed in a temperature-controlled cuvette holder, which was connected via a light guide to a spectrograph (SR-303i-A, Andor, Belfast, UK). For recording the spectra, an ICCD camera (Andor iStar, DH320T-18U-63, Andor, Belfast, UK) was used. The excitation wavelength (∼5 ns pulse, NT230, Ekspla, Vilnius, Lithuania) was 394 nm (grating: 300 mm^−1^). For Cm(III) luminescence spectroscopy, a pulsed flash lamp pumped Nd:YAG laser system (Powerlite Precision II 9020 laser) equipped with a Green PANTHER EX OPO (Continuum, Santa Clara, CA, USA) was used. The laser system was equipped with a delay generator (Model DG535, Stanford Research Systems Inc., Sunnyvale, CA, USA). The luminescence spectra were detected using an optical multichannel analyzer system, consisting of an Kymera 328i monochromator and spectrograph with gratings of 150, 300, 600, and 1200 lines per mm (Oxford Instruments, Abingdon, UK) and an Andor iStar ICCD camera (ICCD 05933, Andor, Belfast, UK). The excitation wavelength was 396 nm (grating: 300 mm^–1^).

### 3.6. Data Processing Software

Speciation calculations of Eu(III) under varying conditions were carried out with PHREEQC Interactive, version 3.7.3-15968. The complexation constants were taken from the Paul Scherrer Institute (PSI)/TDB 2020. Additional data were added from Delgado et al. [93], Friedrich et al. [56,78], and Smith et al. [71,72]. NMR spectra were processed with MestReNova, version 6.0.2., Mestrelab Research S.L., Santiago de Compostela, Spain [94]. The multidimensional TRLFS data obtained under challenging conditions were analyzed using state-of-the-art mathematical tools to distinguish between species with different luminescence properties. Therefore, parallel factor analysis (PARAFAC) was used, as described elsewhere [95,96]. Briefly, PARAFAC is a generalization of principal component analysis (PCA) to higher order arrays. An advantage of applying PARAFAC to three-dimensional data (such as a set of TRLFS data) is that it overcomes the rotational issues inherent in bilinear two-dimensional methods thanks to its trilinearity. As a result, PARAFAC provides a unique and easy to interpret model [97]. Creation of graphs for numerical data visualization and data fitting by a nonlinear sigmoidal dose–response fit algorithm was performed with Origin 2019, version 9.6.0.172, OriginLab Corporation, Northhampton, MA, USA. For the calculation and visualization of ligand affinities, Matplotlib 3.7.1 in Python 3.11.2 was applied [98,99].

## 4. Conclusions

This study investigated the displacement efficacy of EGTA, EDTA, DTPA, and HOPO as (potential) DAs for Eu(III) and Cm(III) in simulated gastrointestinal conditions according to the UBM protocol. The results showed that all tested DAs were able to fully displace bioligands from the coordination spheres of Eu(III) and Cm(III) at sufficient concentrations, with significant differences in their efficacy. Under almost all analyzed conditions, the observed efficacy followed the trend EGTA << EDTA < DTPA ~ HOPO which is in the order of the pH-dependent affinities of the ligands.

These results were obtained under stationary conditions, where parameters such as pH, ligand concentrations, and temperature were kept constant, without the dynamic physiological processes of living organisms, such as absorption, transport, metabolism, and excretion. While this simplifies interpretation, it limits the direct applicability of these results to in vivo conditions. Furthermore, the toxicity of the ligands itself on cells or mammals in general are not considered in this study. Especially EDTA and DTPA have been subject of investigations towards their acute toxicity, showing disadvantageous properties [44]. Nonetheless, the findings provide an essential foundation for understanding DA efficacy in a controlled and reproducible environment.

Importantly, the established methodology enables the estimation of the efficacy of similar ligands based on their thermodynamic properties, in particular stability constants and p*K*_a_ values. This approach facilitates the rational design and selection of new ligands with high affinity for target metals and properties optimized for physiological conditions.

The results emphasize the superior efficacy of acyclic ligands like DTPA and HOPO compared to cyclic DOTA, which allow effective cation exchange—a critical property for overcoming kinetic limitations. This study provides a robust framework for evaluating and optimizing decorporation agents and lays the foundation for developing more effective chelation therapies for lanthanide and actinide contamination, with the potential to bridge the gap to dynamic in vivo systems in future research.

## Figures and Tables

**Table 1:** Composition of the single biofluids and the whole digestive system (gastrointestinal tract, GIT) as given in the modified UBM protocol [51,52].

Chemicals		Saliva (15%)	Gastric Juice (23%)	Pancreatic Juice (46%)	Bile (15%)	GIT
Inorganics	mmol/L					
	NaCl	10.2	94.2	234	180	159
	KCl	24.0	22.1	15.1	10.1	17.3
	NH_4_Cl	-	11.4	-	-	2.63
	MgCl_2_	-	-	0.5	-	0.23
	CaCl_2_	1.0	-	1.4	1.5	2.28
	NaH_2_PO_4_	14.8	3.9	-	-	3.18
	KH_2_PO_4_	-	-	1.2	-	0.55
	NaHCO_3_	-	-	133.5	137.7	82.8
	KHCO_3_	15.0	-	-	-	2.31
	Na_2_SO_4_	8.0	-	-	-	1.23
	KSCN	4.1	-	-	-	0.63
Organics	mmol/L					
	urea	6.7	2.8	3.3	8.3	4.58
	uric acid	0.1	-	-	-	0.02
	glucose	-	7.2	-	-	1.66
	glucosamine∙HCl	-	3.1	-	-	0.72
	glucuronic acid	-	0.2	-	-	0.05
Enzymes	mg/mL					
	α-amylase	1.0	-	-	-	0.15
	mucin	0.5	3.0	3.0	-	2.15
	pepsin	-	1.0	-	-	0.23
	pancreatin	-	-	3.0	-	1.85
	trypsin	-	-	1.0	-	0.46
	lipase	-	-	0.5	-	0.23
	bile extract	-	-	-	6.0	0.92
*I* (mM)		95	289	455	342	344
pH		6.5 ± 0.5	1.0 ± 0.2	7.4 ± 0.2	8.0 ± 0.2	6.5 ± 0.5

**Table 2:** Summarized TRLFS data of Eu(III) and Cm(III) complexes with different biological and chelating ligands from this work and references from the literature.

Species	pH	Lifetime (µs)	^6^D_7/2_ → ^8^S_7/2_ Peak Maximum (nm)	Ref.
Eu(phosphate)	6.5 ± 0.5	207 ± 55	-	this work
	6.4	235 ± 10	-	[52]
	6–7	278 ± 8	-	[69]
Eu(amylase)	5.5	380 ± 40	-	[22]
	6.2	412 ± 21/794 ± 18	-	[52,67]
Eu(lipase)	6.4	269 ± 18/677 ± 11	-	[52]
Eu(mucin)	7.2	311 ± 16/746 ± 16	-	[52]
	7.0	267 ± 8/699 ± 7	-	[67]
Eu(pancreatin)	6.0	314 ± 31/768 ± 11	-	[52]
Eu(GIT) ^a^	6.5 ± 0.5	315 ± 67	-	this work
	6.8	261 ± 11/1299 ± 32	-	[52]
[Eu(EDTA)]^−^	6.5 ± 0.5	326 ± 11	-	this work
	2.0	299 ± 6	-	[56]
[Eu(EGTA)]^−^	6.5 ± 0.5	553 ± 40	-	this work
	3.0	586 ± 5	-	[56]
[Eu(DTPA)]^2−^	6.5 ± 0.5	545 ± 99	-	this work
	6.75 ± 0.25	629	-	[57]
[Eu(HOPO)]^−^	6.5 ± 0.5	713 ± 63	-	this work
	7.4	805 ± 81	-	[58]
Cm(GIT) ^a^	6.5 ± 0.5	152 ± 9	604.5	this work
	6.8	138 ± 7/498 ± 13	603.7/603.7	[52]
Cm(amylase)	5.5	120 ± 10/240 ± 40	598/603	[22]
Cm(mucin)	6.0	81 ± 5/259 ± 5	603.1/603.1	[52]
	7.2	123 ± 6/326 ± 15	603.5/603.5	[67]
[Cm(EDTA)]^−^	6.5 ± 0.5	136 ± 3	604.0	this work
	2.4	137 ± 5	603.7	[56]
[Cm(EGTA)]^−^	6.5 ± 0.5	232 ± 3	609.0	this work
	3.0	262 ± 5	609.1	[56]
[Cm(DTPA)]^2−^	6.5 ± 0.5	233 ± 3	607.5	this work
	-	268	606	[70]
[Cm(HOPO)]^−^	6.5 ± 0.5	260 ± 3	611.8	this work
	7.4	383 ± 38	610	[60]

^a^ Eu(III)/Cm(III) not further specified binding partners from the complete GIT mixture, bound to proteins and phosphates according to ref. [52].

**Table 3:** DC_50_ values of EGTA, EDTA, DTPA, and HOPO for Eu(III) and Cm(III) in different media and temperatures (see Figure 4, Figure 5 and Figure 6).

Media	M(III)	T (°C)	DC_50_ (×[M(III)])			
			EGTA	EDTA	DTPA	HOPO
Phosphate	Eu(III)	25	10.1 ± 1.4	1.1 ± 0.1	0.5 ± 0.1	0.5 ± 0.1
		37	37 ± 19	1.2 ± 0.2	0.3 ± 0.1	0.6 ± 0.1
GIT	Eu(III)	25	106 ± 5	69 ± 29	26 ± 22	1.7 ± 0.5
		37	58 ± 33	18 ± 4	1.9 ± 1.0	1.2 ± 0.2
	Cm(III)	37	1700 ± 170	388 ± 39	75 ± 8	96 ± 10

**Table 4:** Thermodynamic data (*I* = 0.1 M, NaCl/NaClO_4_) and complexing properties of the chosen DA and their corresponding 1:1 complexes with Eu(III), Cm(III), and Ca(II).

	EDTA [56]	EGTA [56]	DTPA [62]	HOPO [58]
p*K*_a1_	1.12 ± 0.06	1.45 ± 0.04	2.12 ± 0.07	3.87 ± 0.01
p*K*_a2_	2.50 ± 0.02	2.28 ± 0.08	2.87 ± 0.03	5.01 ± 0.01
p*K*_a3_	6.10 ± 0.01	9.25 ± 0.01	4.52 ± 0.04	5.68 ± 0.01
p*K*_a4_	9.65 ± 0.01	9.25 ± 0.01	8.75 ± 0.02	6.64 ± 0.01
p*K*_a5_	-	-	10.17 ± 0.03	-
Denticity	6	8	8	8
log *K*_110_ ([EuL]^x−^) ^a^	17.0 ± 0.1	17.9 ± 0.2	22.56 ± 0.14	20.2 ± 0.2
log *K*_110_ ([CmL]^x−^) ^a^	17.5 ± 0.03	18.6 ± 0.01	22.64 ± 0.13	21.8 ± 0.4 [60]
log *K*_110_ ([CaL]^y−^) ^a^	10.5 [71]	11.1 [72]	10.7 [71]	-

^a^ *K_MLH_* where *M*, *L*, and *H* denote the stoichiometric coefficients of metal ion, ligand, and protons involved, respectively. x = 1 for EDTA, EGTA, and HOPO; x = 2 for DTPA; y = 2 for EDTA and EGTA; y = 3 for DTPA.

**Table 5:** Calculated affinities of the four DA towards Eu(III) and Cm(III) at pH 6.5.

M(III)	EGTA	EDTA	DTPA	HOPO
Eu(III)	12.4	13.7	16.6	19.8
Cm(III)	13.1	14.2	16.7	21.4

**Table 6:** Fit parameters for the displacing efficacy depending on the affinities.

Media	M	*T* (°C)	A	B	C
Phosphate	Eu(III)	25	0.50 ± 0.01	(−1.63 ± 1.40) × 10^12^	0.12 ± 0.01
		37	0.56 ± 0.14	(−1.02 ± 8.12) × 10^16^	0.07 ± 0.04
GIT	Eu(III)	25	0.50 ^a^	(−2.03 ± 32.2) × 10^5^	0.54 ± 0.01
		37	1.16 ± 0.04	(−1.55 ± 1.34) × 10^7^	0.37 ± 0.02
	Cm(III)	37	79.5 ± 14.3	(−1.61 ± 7.56) × 10^12^	0.21 ± 0.07

^a^ Value fixed to 0.5 based on metal:ligand stoichiometry in complexes to ensure a fit convergence.

## Data Availability

All data are included in this paper.

## Supplementary Materials

The following supporting information can be downloaded at: https://www.mdpi.com/article/10.3390/ijms26157112/s1.

## Abbreviations

The following abbreviations are used in this manuscript:

Ln	Lanthanides
An	Actinides
EDTA	Ethylenediaminetetraacetic acid
EGTA	Ethylene glycol-bis(β-aminoethyl ether)-*N,N,N′,N′*-tetraacetic acid
DTPA	Diethylenetriaminepentaacetic acid
HOPO	Spermine-based hydroxypyridonate chelator 3,4,3-LI(1,2-HOPO)
UBM	Unified bioaccessibility method
TRLFS	Time-resolved laser-induced fluorescence spectroscopy
NMR	Nuclear magnetic resonance
RN	Radionuclides
DA	Decorporation agents
GIT	Gastrointestinal tract
DC_50_	Decorporation concentration for 50% displacement
PARAFAC	Parallel factor analysis
