# Supplementary material for: Efficacy of Various Complexing Agents for Displacing Biologically Important Ligands from Eu(III) and Cm(III) Complexes in Artificial Body Fluids—An In Vitro Decorporation Study
Source: Int J Mol Sci. 2025 Jul 23;26(15):7112. doi: 10.3390/ijms26157112 (PMC12346176; doi:10.3390/ijms26157112)
Supplement: Supplementary file 1 [file ijms-26-07112-s001.zip › ijms-3725951-supplementary.pdf]

Supporting Information

Efficacy of Various Complexing Agents for Displacing Biologically Important Ligands from Eu(III) and Cm(III) Complexes in Artificial Body Fluids—An In Vitro Decorporation Study

Sebastian Friedrich [1], Antoine Barberon [2], Ahmadabdurahman Shamoun [3], Björn Drobot [1], Katharina Müller [1], Thor-sten Stumpf [1], Jerome Kretzschmar [1] and Astrid Barkleit [1,*]

[1] Helmholtz-Zentrum Dresden-Rossendorf, Institute of Resource Ecology, 01328 Dresden, Germany; s.friedrich@hzdr.de (S.F.); b.drobot@hzdr.de (B.D.); k.mueller@hzdr.de (K.M.); t.stumpf@hzdr.de (T.S.); j.kretzschmar@hzdr.de (J.K.)
[2] National School of Chemistry Montpellier, 34090 Montpellier, France; antoine.barberon@enscm.fr
[3] Institute of Radioecology and Radiation Protection, Leibniz University Hannover, 30419 Hannover, Germany; shamoun@irs.uni-hannover.de
* Correspondence: a.barkleit@hzdr.de

Supporting Information Contents

Synthesis of 3,4,3-LI(1,2-HOPO):

HOPO synthesis scheme **(Scheme S1)** and experimental details S3-S4

HOPO ESI-MS, NMR, FT-IR spectra (**Figure S1**) S5

Time-resolved Laser-induced fluorescence spectroscopy with Eu(III):

Phosphate displacement by EGTA (**Figures S2, S3, Tables S1, S2**) S6-S7

Phosphate displacement by EDTA (**Figures S4, S5, Tables S3, S4**) S8-S9

Phosphate displacement by DTPA (**Figures S6, S7, Tables S5, S6**) S10-S11

Phosphate displacement by HOPO (**Figures S8, S9, Tables S7, S8**) S12-S13

Displacement of all GIT components by EGTA (**Figures S10, S11, Tables S9, S10**) S14-S15

Displacement of all GIT components by EDTA (**Figures S12, S13, Tables S11, S12**) S16-S17

Displacement of all GIT components by DTPA (**Figures S14, S15, Tables S13, S14**) S18-S19

Displacement of all GIT components by HOPO (**Figures S16, S17, Tables S15, S16**) S20-S21

Displacement of all GIT components by DOTA (**Figure S18**) S22

Time-resolved Laser-induced fluorescence spectroscopy with Cm(III):

Displacement of all GIT components by EGTA, EDTA, DTPA, HOPO (**Figure S19**) S22

Thermodynamic modelling with Eu(III):

Displacement of all GIT components by EGTA (**Figure S20, Table S17**) S23-S24

Displacement of all GIT components by EDTA (**Figure S21, Table S18**) S25-S26

Displacement of all GIT components by DTPA (**Figure S22, Table S19**) S27-S28

Displacement of all GIT components by HOPO (**Figure S23, Table S20**) S29

Displacement of all GIT components by DOTA (**Figure S24, Table S21**) S30-S31

Displacement of all GIT components by DEGTA (**Figure S25, Table S22**) S32

NMR spectroscopy:

^{2}H-NMR spectra EGTA-d_8 / GIT / Eu(III) (**Figures S26-S33, Table S23**) S33-S37

Photographs:

Artificial GIT solutions (**Figure S34**) S37

1 Synthesis of 3,4,3-LI(1,2-HOPO)

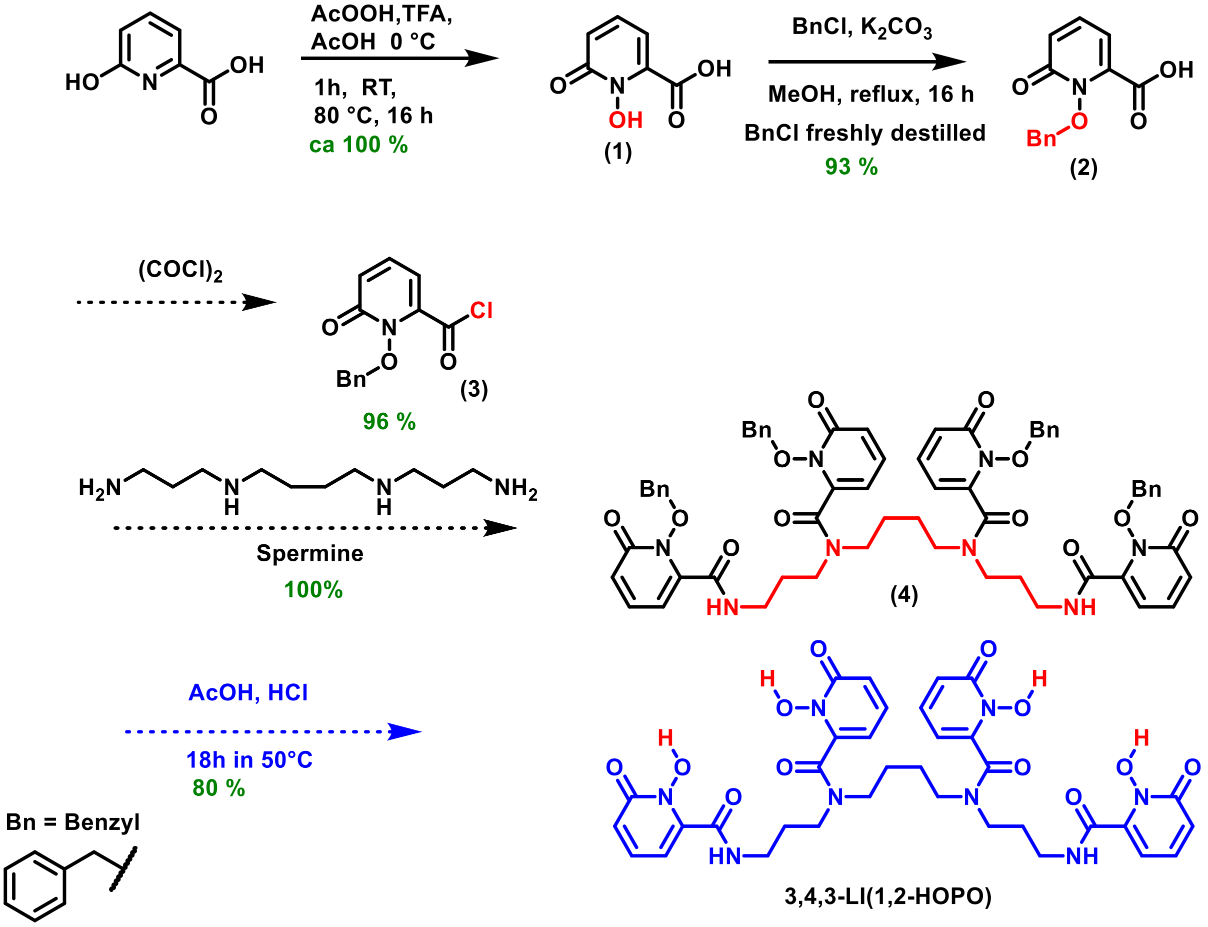

Scheme S1: Synthesis of 3,4,3-LI(1,2-HOPO). Reagents and conditions: a) CH_3COOOH (1.2 equiv), AcOH, TFA, 1 h, 100%; b) BnCl (1.2 equiv), K_2CO_3 (2.0 equiv), CH_3OH, 16 h, 63 °C, 93%; c) $(COCl)_2$ (7.00 equiv), CH_2Cl_2, 0-25 °C, 6 h 96%; d) spermine (0.24 equiv), TEA (1.0 equiv), CH_2Cl_2, 0-25 °C, 24 h, 100% over steps c & d; e) AcOH, HCl (12 N), 18 h, 45 °C, 80%.

6-Carboxy-1-hydroxy-2(1H)-pyridinone [1,2-HOPO carboxylic acid] (1):

The 6-Hydroxypicolinic acid (20.0 g, 143.77 mmol) and peracetic acid 38-40% (46.04 g, 230.03 mmol) were added to a stirring solution of glacial acetic acid (120.0 mL) and trifluoroacetic acid (200.0 mL). This mixture was stirred under nitrogen for 1 h at room temperature before being heated to 80 °C for 12 h. The reaction mixture started off a red-brown color and turned orange over time. A light color precipitate formed, was collected by filtration, and washed with cold methanol. After drying, the product was obtained as a beige solid (yield 100%).

1-Benzyloxy-6-carboxy-2(1H)-pyridinone [1,2-HOPOBn carboxylic acid] (2):

Compound 1 (12.72 g, 82.01 mmol) and potassium carbonate (22.67 g, 164.01 mmol) were added to a stirring solution of benzyl chloride (12.46 g, 98.41 mmol) in methanol (205.016 mL). The mixture was heated to 63 °C and left to reflux for 16 h. The reaction mixture was a dark green suspension and formed a precipitate over time. The blue-gray solid was filtered out of the reaction mixture and the filtrate was collected. The solvent was evaporated and its residue was redissolved in 48.0 mL water. The solution was acidified to pH 2 by dropwise addition of 12 N HCl. An off-white precipitate was formed and was isolated by filtration. The product was washed with cold water and dried to yield a light beige solid (yield 93%).

1-Benzyloxy-2(1H)-pyridinone-6-carbonyl chloride [1,2-HOPOBn acid chloride] (3):

A suspension of 2 (5.00 g, 20.39 mmol) in anhydrous dichloromethane (129.38 mL) was cooled to 0 °C. Excess oxalyl chloride (12.24 mL, 142.72 mmol) was added while stirring followed by catalytic amount of DMF (0.005 mL). Gas bubbles evolved, and the suspension became clear. The mixture was then left at room temperature for 6 h, and the solvent was removed by rotary evaporation to leave pale brown oil. After the oil was co-evaporated twice with toluene (10 mL), the crude acid chloride (3) was used directly for the next reaction without any purification (yield 96%).

3,4,3-(LI-1,2-HOPO)Bn [benzyl-protected HOPO] (4):

A solution of crude 3 (5.00 g, 18.96 mmol) in dichloromethane (225.74 mL) was added drop wise to a stirred solution of triethylamine (2.89 mL, 20.86 mmol), spermine (0.9135 g, 4.51 mmol) and DMAP (0.116 g, 0.95 mmol) in dry dichloromethane (12.60 mL) at 0 °C. The reaction mixture was warmed to room temperature and stirring was continued for 24 h. The reaction mixture was washed with 10% $NaHCO_3$ solution, followed by water. The organic phase was dried over anhydrous Na_2SO_4, filtered, and concentrated to dryness. Purification by column chromatography (SiO_2, 2-6% MeOH in CH_2Cl_2) afforded the benzyl-protected precursor 3,4,3-LI(1,2-HOPOBn) (4) as white foam (yield 100%).

3,4,3-LI(1,2-HOPO) (HOPO):

Compound 4 (0.44 g, 0.40 mmol) was dissolved in 1:1 mixture of acetic acid and concentrated HCl (15 mL) at room temperature and the reaction mixture was stirred at 50 °C for 18 h. The reaction progress was monitored by TLC. The crude product was dried, redissolved in water, and purified by HPLC on a semi-preparative C4 column (Jupiter 250 × 10 mm, 5 µM, Phenomenox) using a gradient of 5-28% MeCN in water (both containing 0.1% TFA) over 20 min. The product peak was collected from 7-8.92 min and the eluted solution was lyophilized to recover the product as a white solid. The purified ligand was collected in multiple small batches with an approximate combined yield of 80%.

The ligand has been characterized by ESI-MS, ^1H NMR and FT-IR spectroscopy (see Figure S1). Consistent with the results from ESI-MS and NMR spectroscopy, the IR spectrum mirrors key features associated with the ligand structure. Bands observed at 3401 and 3268 cm^{-1} correspond to OH and NH stretching vibrations associated with the (CO)NH linkages and (CO)NOH functional groups. The band at 3084 cm^{-1} is characteristic for the CH(sp^2) stretching vibration of the hydroxypyridinone CH=CH fragments, while the CH(sp^3) antisymmetric and symmetric stretching vibrations of the backbone CH_2 groups give rise to the bands detected at 2944 and 2874 cm^{-1}, respectively. Features at 1649, 1573, and 1469 cm^{-1} are due to the C=O stretching vibrations arising from the CO(NOH) functional groups and CO(NH) linkages, likely overlapping with C=C and C–C stretching vibrations. The bands at 1169 and 805 cm^{-1} are tentatively assigned to the N–O stretching vibration of the (CO)NOH moiety and to the NH out-of-plane deformation vibration, respectively.

All spectra are in very good accordance with those reported previously (Deri, M.A., et al., J. Med. Chem., 2014, 57, 4849-4860; DOI: 10.1021/jm500389b), confirming the identity of the ligand.

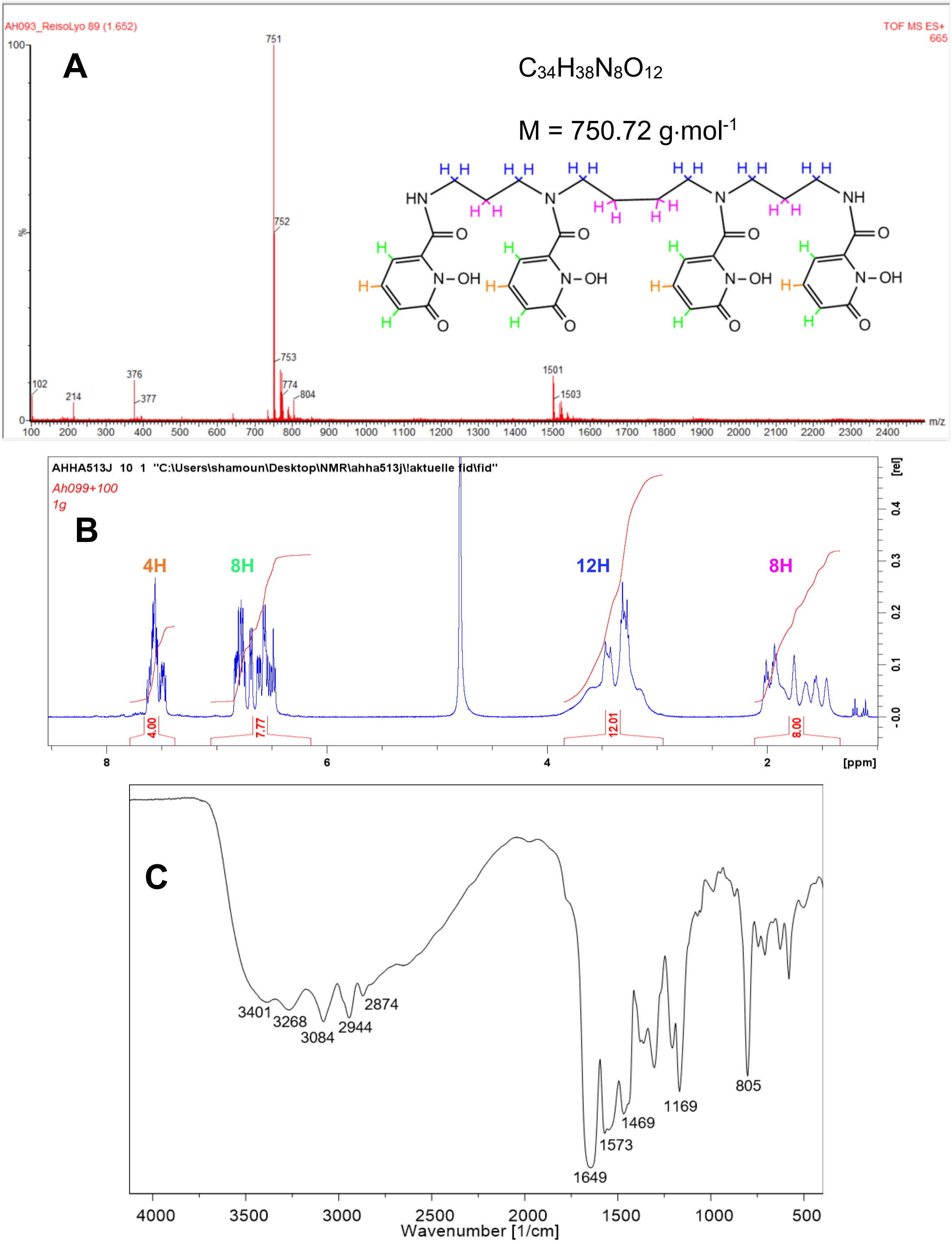

Figure S1: Characterization of 3,4,3-LI(1,2-HOPO). **A**: ESI-MS; **B**: ^{1}H-NMR; **C:** FT IR (KBr Pellet) of the HOPO ligand obtained as white solid after lyophilization of a corresponding aqueous solution (pH 3.5)

2 Time-resolved laser-induced fluorescence spectroscopy

2.1 Displacement of the phosphate fraction of the GIT

2.1.1 EGTA

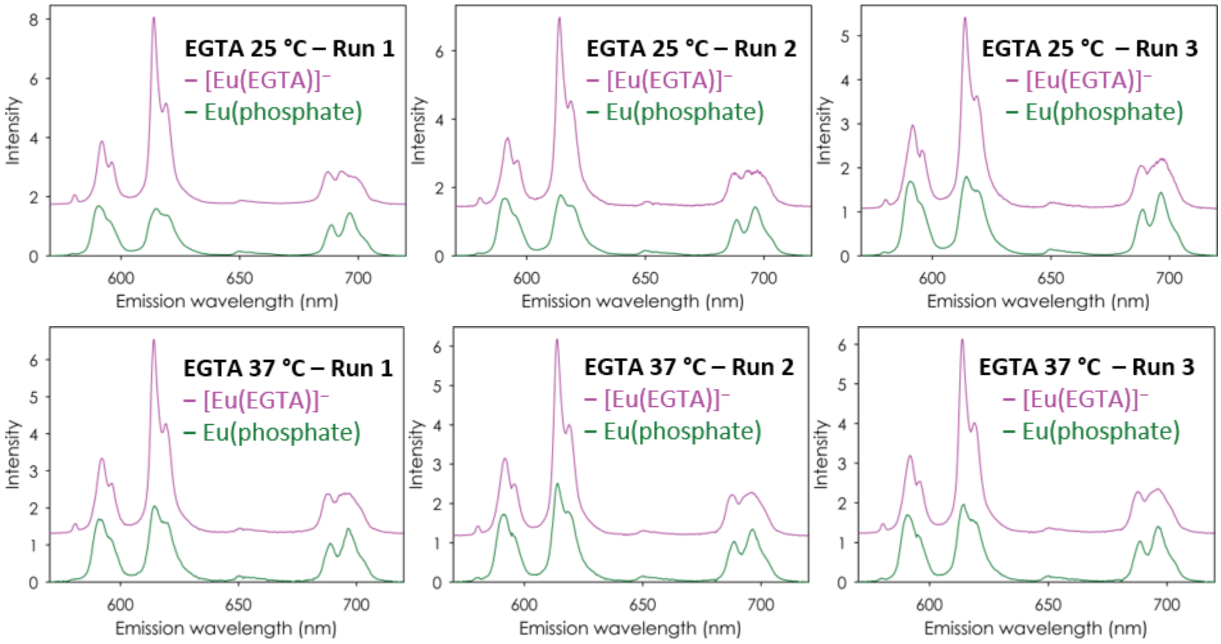

Figure S2: Emission spectra of $[Eu(EGTA)]^-$ and europium(III) phosphate extracted from three different experiments at varying EGTA concentrations using PARAFAC. T = 25 °C (top row) or 37 °C (bottom row), [Eu(III)] = 10 µM, [phosphate] = 3.73 mM, I (NaCl) = 344 mM, pH = 6.5 ± 0.5.

Table S1: Excitation decay lifetimes of $[Eu(EGTA)]^-$ and europium(III) phosphate extracted from three different experiments at varying EGTA concentrations using PARAFAC. T = 25 °C (top row) or 37 °C (bottom row), [Eu(III)] = 10 µM, [phosphate] = 3.73 mM, I (NaCl) = 344 mM, pH = 6.5 ± 0.5.

**25 °C**			
Run	1	2	3
lifetime (µs) ($[Eu(EGTA)]^-$)	532 ± 15	495 ± 8	513 ± 13
lifetime (µs) (Eu(phosphate))	239 ± 6	233 ± 12	189 ± 13
**37 °C**			
Run	1	2	3
lifetime (µs) ($[Eu(EGTA)]^-$)	539 ± 2	509 ± 3	611 ± 15
lifetime (µs) (Eu(phosphate))	211 ± 4	191 ± 6	135 ± 8

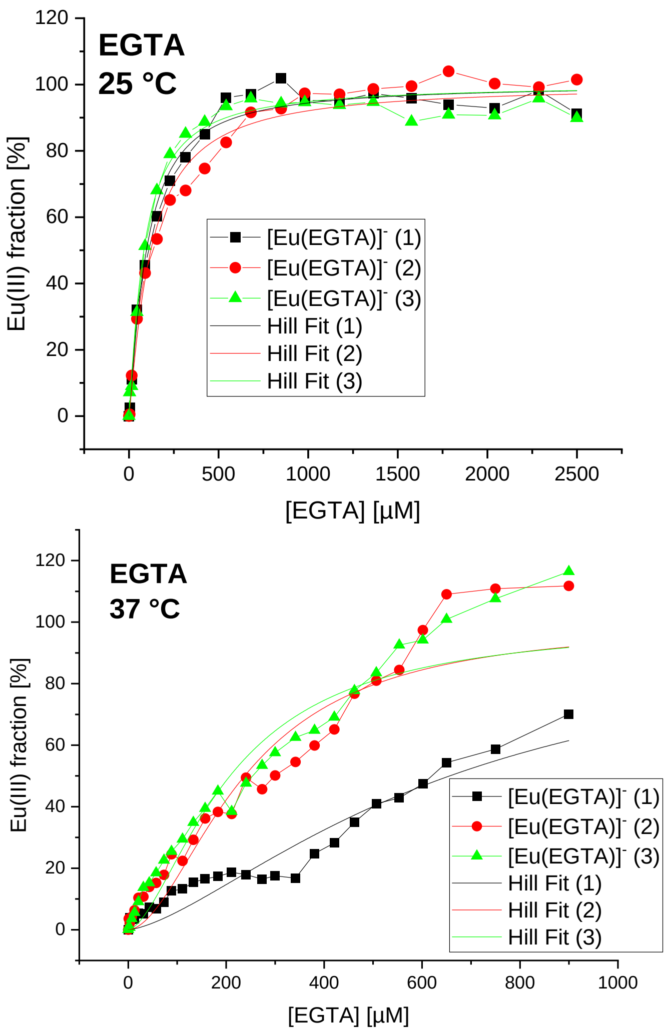

Figure S3: Fractions of the $[Eu(EGTA)]^-$ complex as function of the EGTA concentration and the corresponding Hill fits.

Table S2: Parameters obtained from Hill fit for EGTA at 25 °C and 37 °C

**25 °C**			
Run	1	2	3
k	82.93	121.91	97.37
n	1.17	1.17	1.22
**37 °C**			
Run	1	2	3
k	207.86	237.77	656.77
n	13.81	1.83	1.48

2.1.2 EDTA

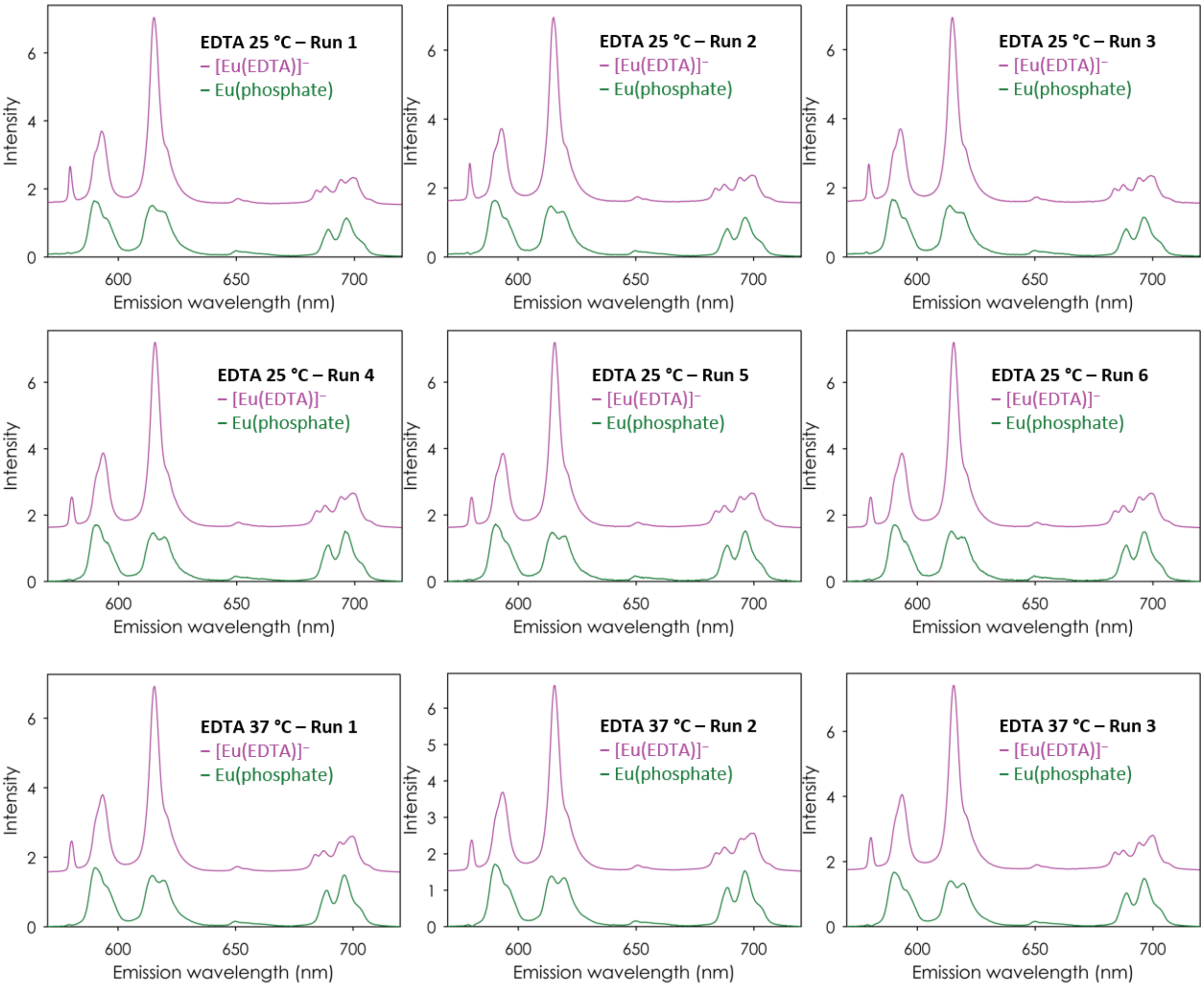

Figure S4: Emission spectra of $[Eu(EDTA)]^-$ and europium(III) phosphate extracted from atleast three different experiments at varying EDTA concentrations using PARAFAC. T = 25 °C (top and middle row) or 37 °C (bottom row), [Eu(III)] = 10 μM, [phosphate] = 3.73 mM, I (NaCl) = 344 mM, pH = 6.5 ± 0.5.

Table S3: Excitation decay lifetimes of $[Eu(EDTA)]^-$ and europium(III) phosphate extracted from atleast three different experiments at varying EDTA concentrations using PARAFAC. T = 25 °C (top row) or 37 °C (bottom row), [Eu(III)] = 10 μM, [phosphate] = 3.73 mM, I (NaCl) = 344 mM, pH = 6.5 ± 0.5.

**25 °C**						
Run	1	2	3	4	5	6
lifetime (μs) ($[Eu(EDTA)]^-$)	319 ± 10	319 ± 5	316 ± 8	324 ± 1	327 ± 2	327 ± 1
lifetime (μs) (Eu(phosphate))	228 ± 16	262 ± 12	247 ± 9	199 ± 3	178 ± 3	186 ± 4
**37 °C**						
Run	1	2	3	-	-	-
lifetime (μs) ($[Eu(EDTA)]^-$)	333 ± 4	324 ± 2	332 ± 6	-	-	-
lifetime (μs) (Eu(phosphate))	220 ± 11	238 ± 15	347 ± 16	-	-	-

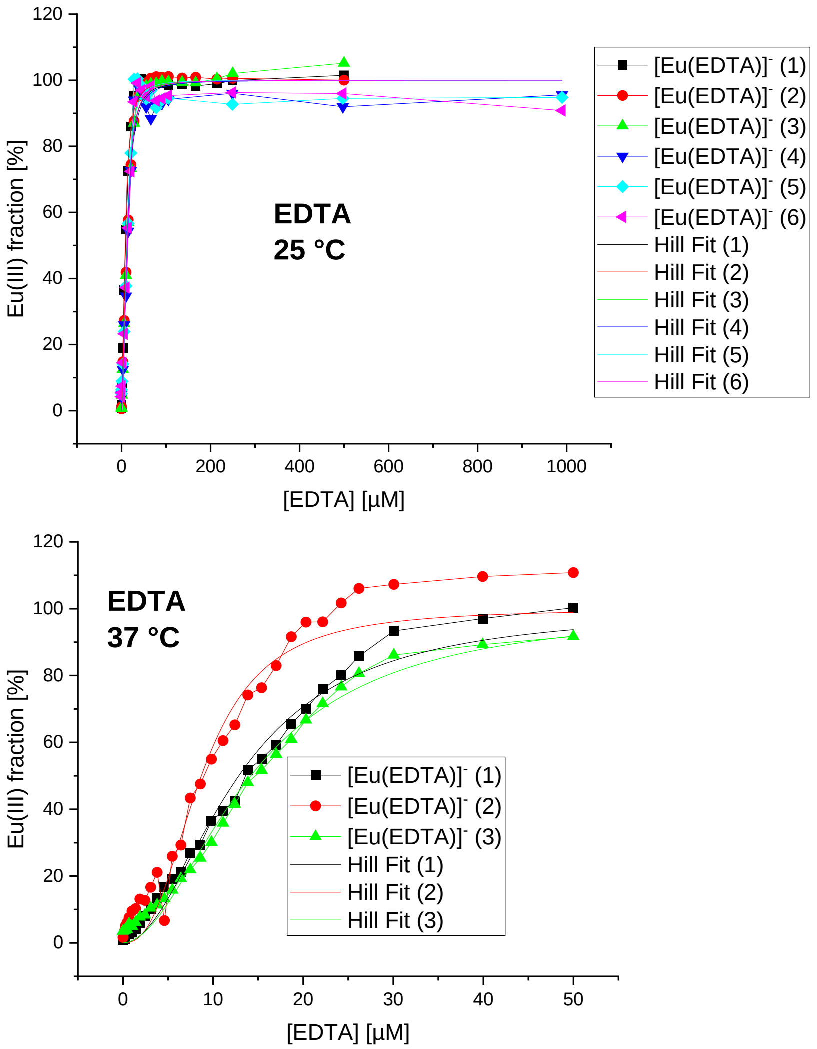

Figure S5: Fractions of the [Eu(EDTA)]⁻ complex as function of the EDTA concentration and the corresponding Hill fits.

Table S4: Parameters obtained from Hill fit for EDTA at 25 °C and 37 °C

			**25 °C**			
Run	1	2	3	4	5	6
k	8.34	11.41	11.78	12.31	11.75	123.23
n	1.88	1.96	1.96	1.91	2.21	2.06
			**37 °C**			
Run	1	2	3	-	-	-
k	12.87	8.71	14.20	-	-	-
n	1.99	2.57	1.92	-	-	-

2.1.3 DTPA

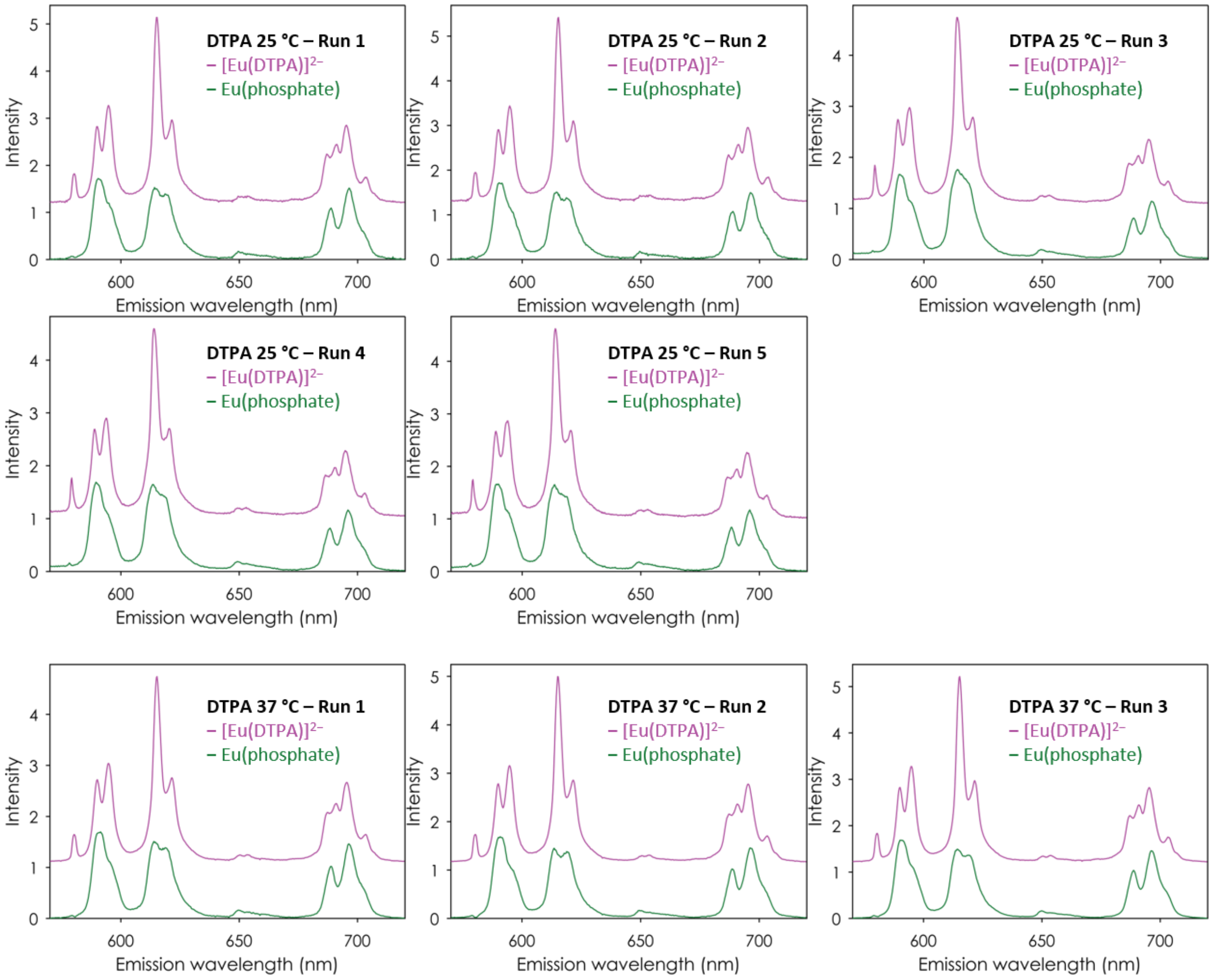

Figure S6: Emission spectra of $[Eu(DTPA)]^{2-}$ and europium(III) phosphate extracted from atleast three different experiments at varying DTPA concentrations using PARAFAC. T = 25 °C (top and middle row) or 37 °C (bottom row), [Eu(III)] = 10 μM, [phosphate] = 3.73 mM, I (NaCl) = 344 mM, pH = 6.5 ± 0.5.

Table S5: Excitation decay lifetimes of $[Eu(DTPA)]^{2-}$ and europium(III) phosphate extracted from atleast three different experiments at varying DTPA concentrations using PARAFAC. T = 25 °C (top row) or 37 °C (bottom row), [Eu(III)] = 10 μM, [phosphate] = 3.73 mM, I (NaCl) = 344 mM, pH = 6.5 ± 0.5.

**25 °C**					
Run	1	2	3	4	5
lifetime (μs) ($[Eu(DTPA)]^{2-}$)	592 ± 2	639 ± 5	461 ± 14	467 ± 10	462 ± 15
lifetime (μs) (Eu(phosphate))	178 ± 2	167 ± 3	183 ± 8	188 ± 4	202 ± 5
**37 °C**					
Run	1	2	3	-	-
lifetime (μs) ($[Eu(DTPA)]^{2-}$)	560 ± 3	603 ± 3	628 ± 2	-	-
lifetime (μs) (Eu(phosphate))	190 ± 3	242 ± 2	334 ± 1	-	-

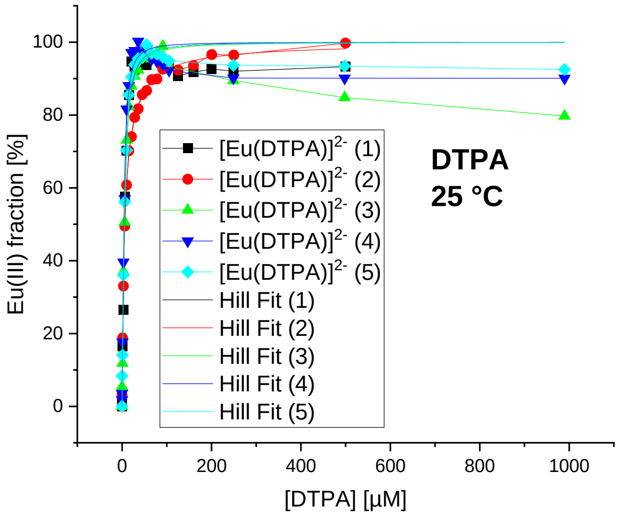

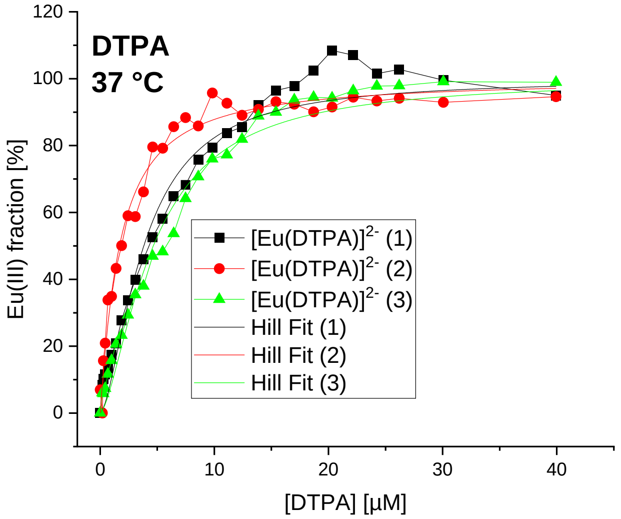

Figure S7: Fractions of the $[Eu(DTPA)]^{2-}$ complex as function of the DTPA concentration and the corresponding Hill fits.

Table S6: Parameters obtained from Hill fit for DTPA at 25 °C and 37 °C

**25 °C**					
Run	1	2	3	4	5
k	6.48	5.23	4.87	5.17	4.22
n	0.91	1.39	1.36	1.31	1.47
**37 °C**					
Run	1	2	3	-	-
k	3.83	1.69	4.66	-	-
n	1.60	1.11	1.53	-	-

2.1.4 HOPO

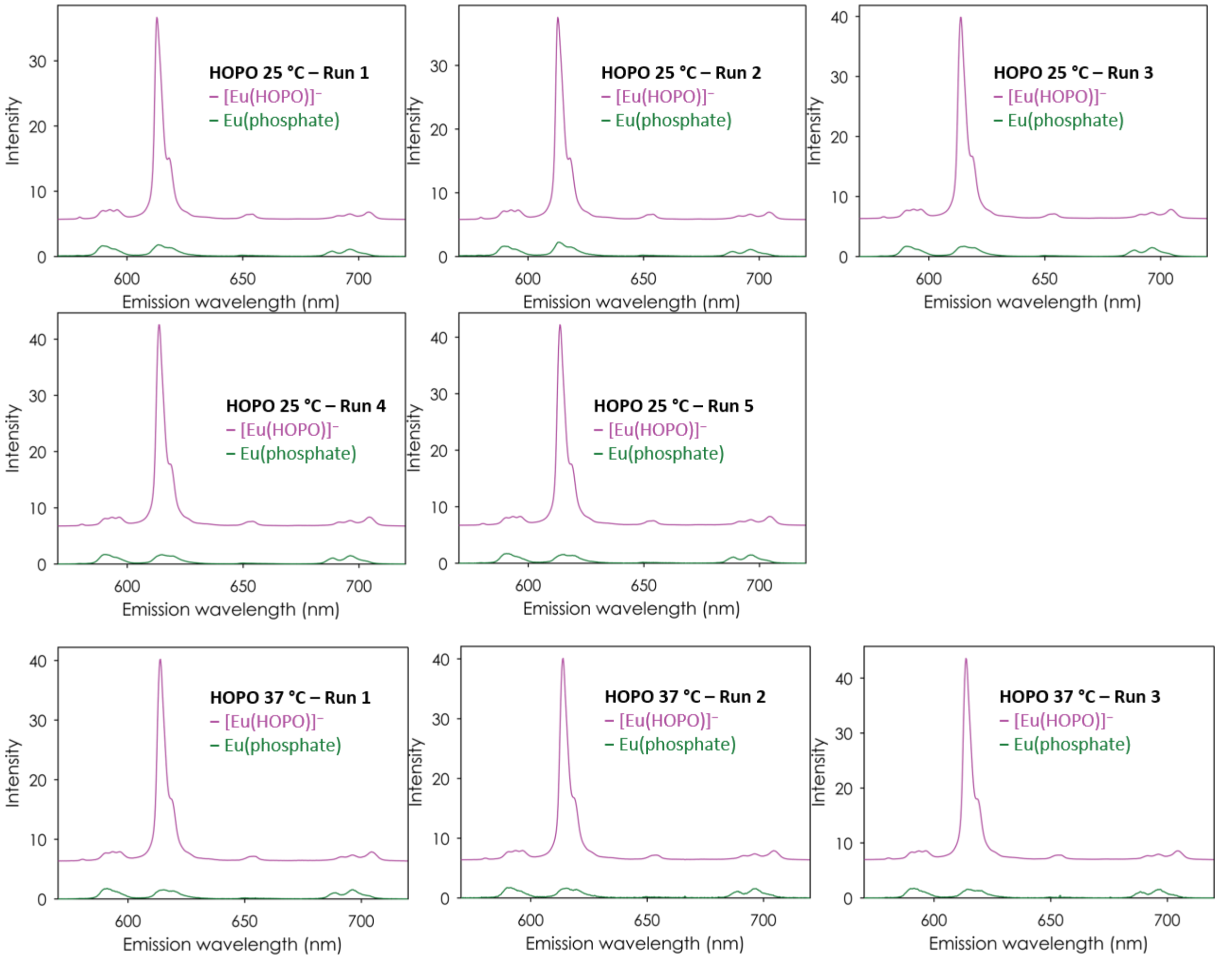

Figure S8: Emission spectra of $[Eu(HOPO)]^{-}$ and europium(III) phosphate extracted from atleast three different experiments at varying HOPO concentrations using PARAFAC. T = 25 °C (top and middle row) or 37 °C (bottom row), [Eu(III)] = 10 μM, [phosphate] = 3.73 mM, I (NaCl) = 344 mM, pH = 6.5 ± 0.5.

Table S7: Excitation decay lifetimes of $[Eu(HOPO)]^{-}$ and europium(III) phosphate extracted from atleast three different experiments at varying HOPO concentrations using PARAFAC. T = 25 °C (top row) or 37 °C (bottom row), [Eu(III)] = 10 μM, [phosphate] = 3.73 mM, I (NaCl) = 344 mM, pH = 6.5 ± 0.5.

**25 °C**					
Run	1	2	3	4	5
lifetime (μs) ($[Eu(HOPO)]^{-}$)	625 ± 15	629 ± 20	772 ± 4	778 ± 3	778 ± 5
lifetime (μs) (Eu(phosphate))	259 ± 17	212 ± 11	241 ± 2	236 ± 2	170 ± 4
**37 °C**					
Run	1	2	3	-	-
lifetime (μs) ($[Eu(HOPO)]^{-}$)	787 ± 4	782 ± 3	787 ± 4	-	-
lifetime (μs) (Eu(phosphate))	118 ± 3	108 ± 5	94 ± 4	-	-

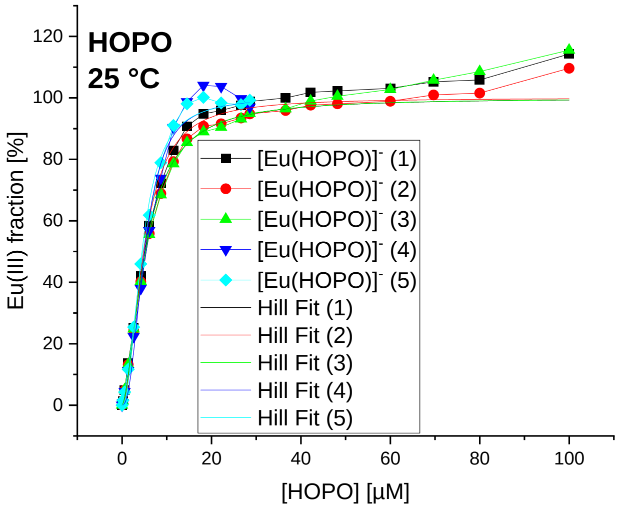

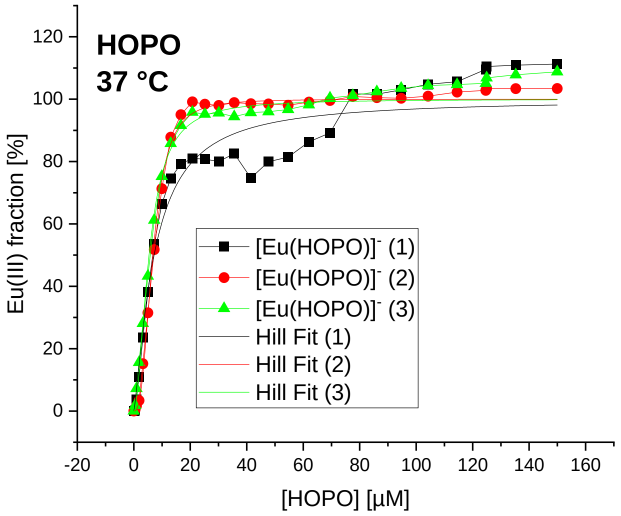

Figure S9: Fractions of the [Eu(HOPO)]$^-$ complex as function of the HOPO concentration and the corresponding Hill fits.

Table S8: Parameters obtained from Hill fit for HOPO at 25 °C and 37 °C

**25 °C**					
Run	1	2	3	4	5
k	5.18	4.85	5.22	5.02	4.48
n	1.68	1.91	1.69	2.37	2.15
**37 °C**					
Run	1	2	3	-	-
k	7.20	6.77	5.42	-	-
n	1.30	2.70	1.87	-	-

2.2 Displacement of all components of the GIT

2.2.1 EGTA

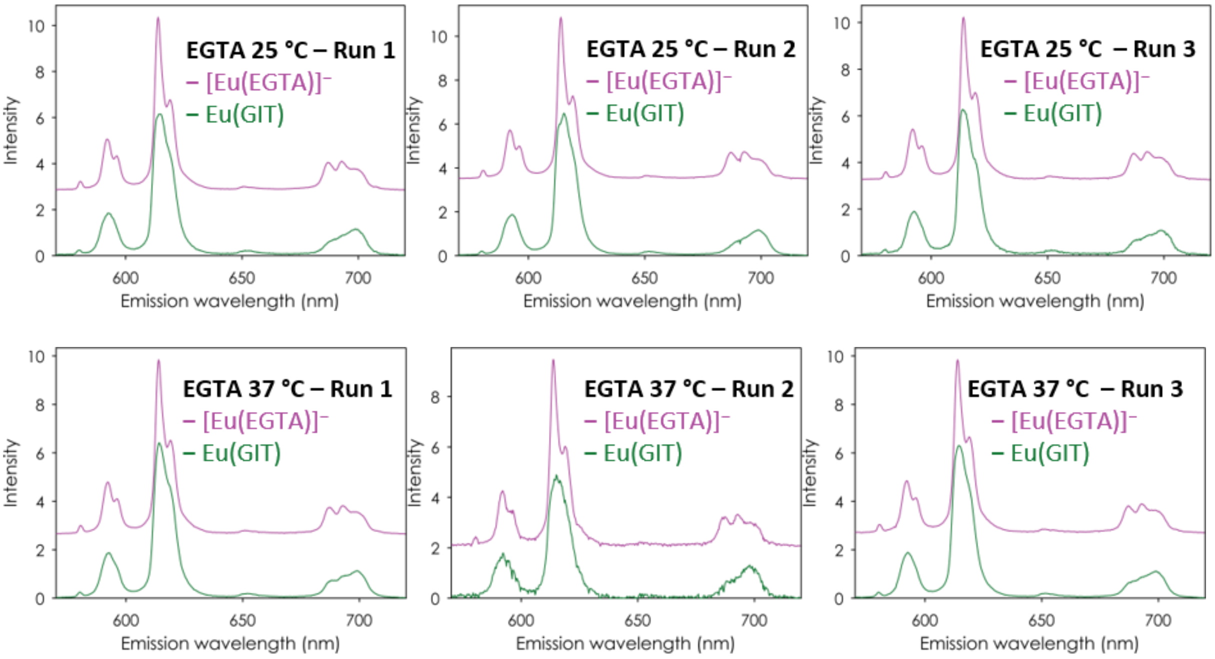

Figure S10: Emission spectra of $[Eu(EGTA)]^-$ and species formed between Eu(III) and components of GIT extracted from three different experiments at varying EGTA concentrations using PARAFAC. T = 25 °C (top row) or 37 °C (bottom row), [Eu(III)] = 10 μM, pH = 6.5 ± 0.5, complete GIT.

Table S9: Excitation decay lifetimes of $[Eu(EGTA)]^-$ and species formed between Eu(III) and components of GIT extracted from three different experiments at varying EGTA concentrations using PARAFAC. T = 25 °C (top row) or 37 °C (bottom row), [Eu(III)] = 10 μM, pH = 6.5 ± 0.5, complete GIT.

**25 °C**			
Run	1	2	3
lifetime (μs) ($[Eu(EGTA)]^-$)	588 ± 3	600 ± 3	550 ± 8
lifetime (μs) (Eu(GIT))	337 ± 5	376 ± 2	356 ± 6
**37 °C**			
Run	1	2	3
lifetime (μs) ($[Eu(EGTA)]^-$)	546 ± 7	541 ± 7	611 ± 17
lifetime (μs) (Eu(GIT))	369 ± 4	120 ± 13	327 ± 2

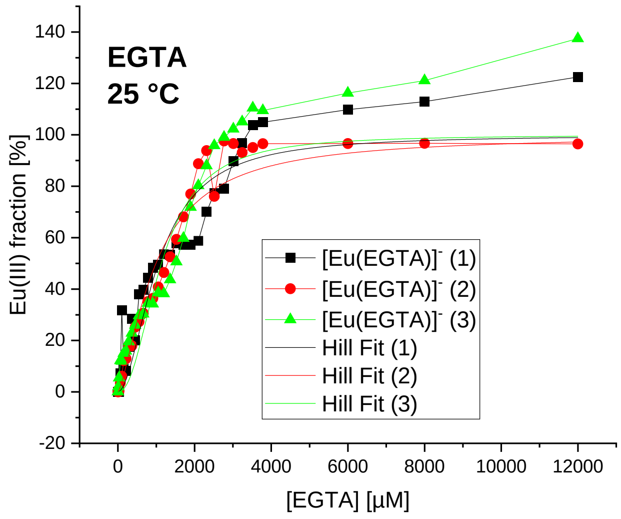

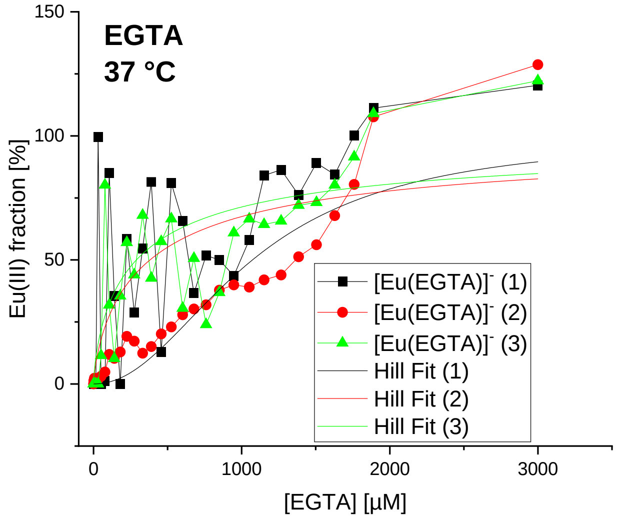

Figure S11: Fractions of the $[Eu(EGTA)]^-$ complex as function of the EGTA concentration and the corresponding Hill fits.

Table S10: Parameters obtained from Hill fit for EGTA at 25 °C and 37 °C

**25 °C**			
Run	1	2	3
k	1056.29	993.43	1136.45
n	1.86	1.43	2.20
**37 °C**			
Run	1	2	3
k	1073.71	381.76	289.96
n	2.09	0.76	0.74

2.2.2 EDTA

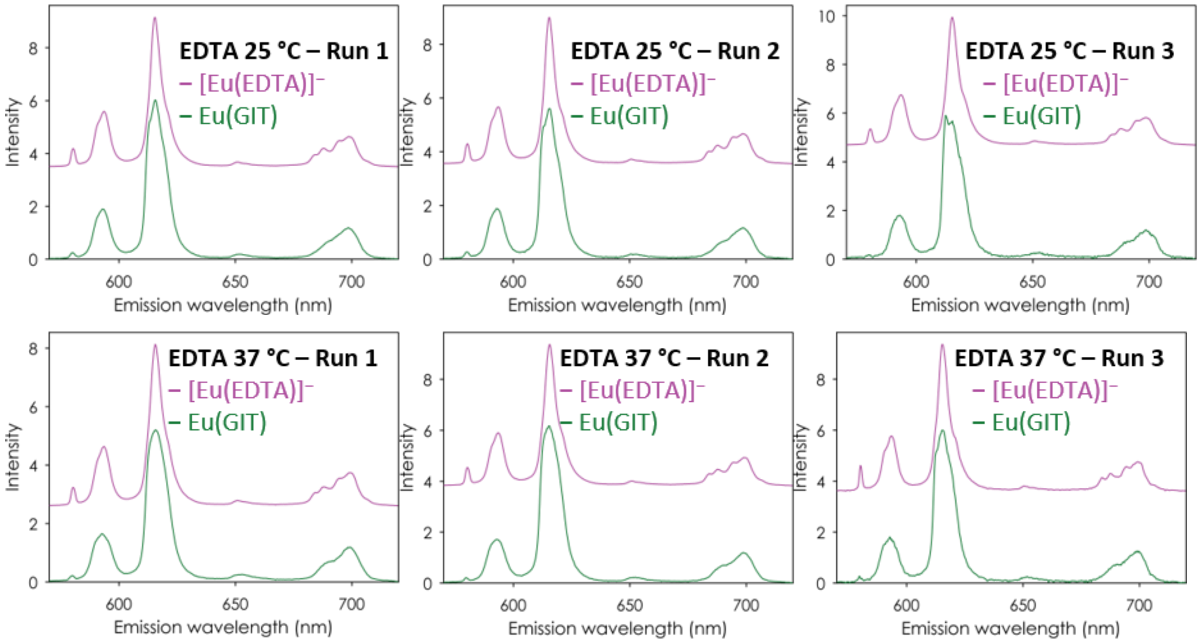

Figure S12: Emission spectra of $[Eu(EDTA)]^-$ and species formed between Eu(III) and components of GIT extracted from three different experiments at varying EDTA concentrations using PARAFAC. T = 25 °C (top row) or 37 °C (bottom row), [Eu(III)] = 10 µM, pH = 6.5 ± 0.5, complete GIT.

Table S11: Excitation decay lifetimes of $[Eu(EDTA)]^-$ and species formed between Eu(III) and components of GIT extracted from three different experiments at varying EDTA concentrations using PARAFAC. T = 25 °C (top row) or 37 °C (bottom row), [Eu(III)] = 10 µM, pH = 6.5 ± 0.5, complete GIT.

**25 °C**			
Run	1	2	3
lifetime (µs) ($[Eu(EDTA)]^-$)	353 ± 2	338 ± 2	332 ± 2
lifetime (µs) (Eu(GIT))	349 ± 3	321 ± 2	270 ± 8
**37 °C**			
Run	1	2	3
lifetime (µs) ($[Eu(EDTA)]^-$)	317 ± 2	303 ± 2	332 ± 2
lifetime (µs) (Eu(GIT))	227 ± 8	261 ± 5	281 ± 3

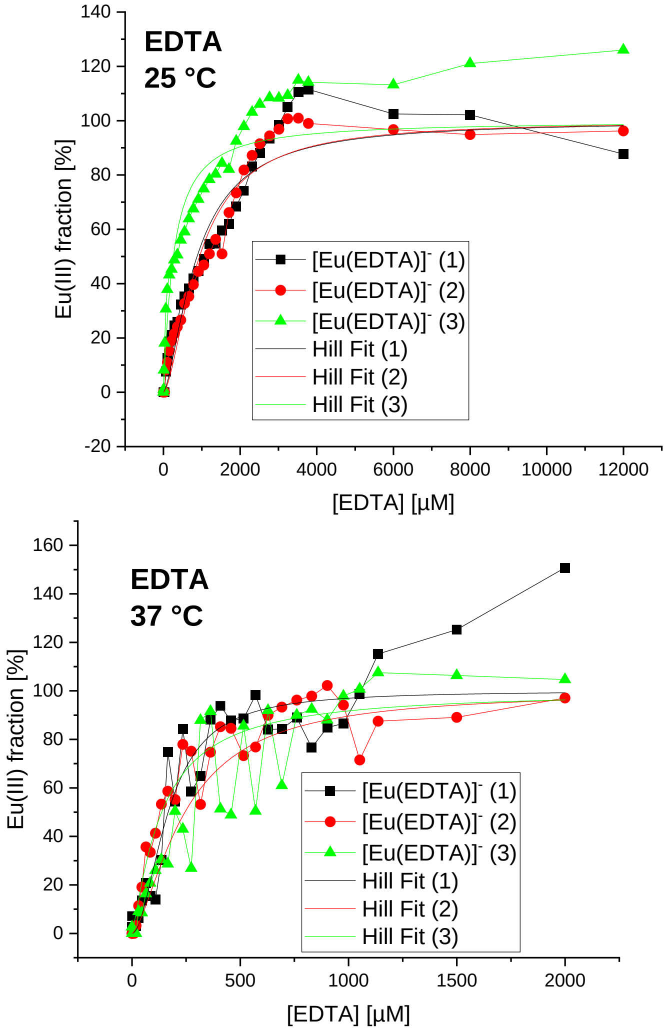

Figure S13: Fractions of the $[Eu(EDTA)]^-$ complex as function of the EDTA concentration and the corresponding Hill fits.

Table S12: Parameters obtained from Hill fit for EDTA at 25 °C and 37 °C

**25 °C**			
Run	1	2	3
k	941.79	259.72	877.66
n	1.60	1.10	1.50
**37 °C**			
Run	1	2	3
k	167.35	244.90	137.59
n	1.93	1.53	1.21

2.2.3 DTPA

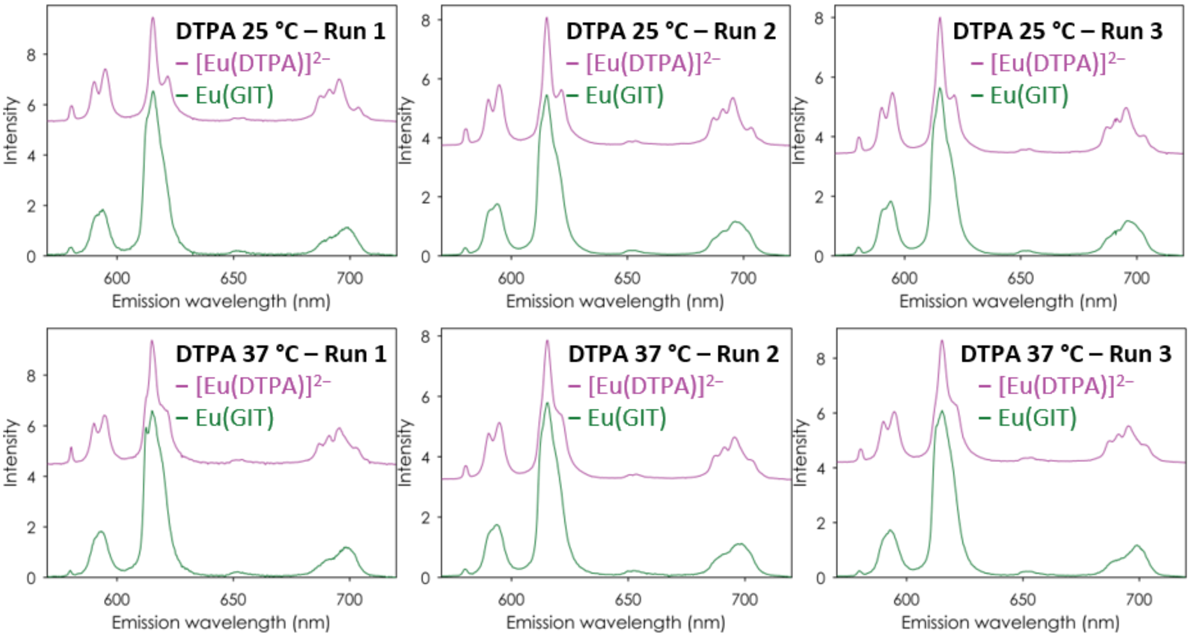

Figure S14: Emission spectra of $[Eu(DTPA)]^{2-}$ and species formed between Eu(III) and components of GIT extracted from three different experiments at varying DTPA concentrations using PARAFAC. *T* = 25 °C (top row) or 37 °C (bottom row), [Eu(III)] = 10 µM, pH = 6.5 ± 0.5, complete GIT.

Table S13: Excitation decay lifetimes of $[Eu(DTPA)]^{2-}$ and species formed between Eu(III) and components of GIT extracted from three different experiments at varying DTPA concentrations using PARAFAC. *T* = 25 °C (top row) or 37 °C (bottom row), [Eu(III)] = 10 µM, pH = 6.5 ± 0.5, complete GIT.

**25 °C**			
Run	1	2	3
lifetime (µs) ($[Eu(DTPA)]^{2-}$)	514 ± 5	608 ± 5	578 ± 4
lifetime (µs) (Eu(GIT))	282 ± 5	399 ± 3	405 ± 3
**37 °C**			
Run	1	2	3
lifetime (µs) ($[Eu(DTPA)]^{2-}$)	457 ± 2	719 ± 230	342 ± 36
lifetime (µs) (Eu(GIT))	335 ± 8	230 ± 26	221 ± 21

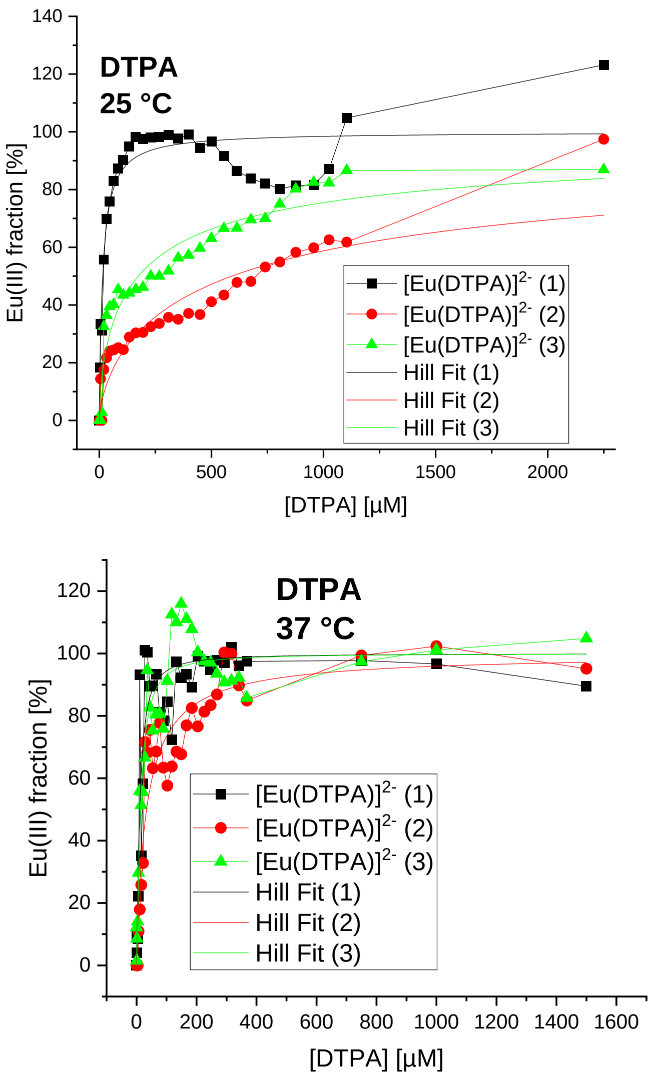

Figure S15: Fractions of the $[Eu(DTPA)]^{2-}$ complex as function of the DTPA concentration and the corresponding Hill fits.

Table S14: Parameters obtained from Hill fit for DTPA at 25 °C and 37 °C

**25 °C**			
Run	1	2	3
k	15.40	175.04	592.21
n	0.99	0.64	0.67
**37 °C**			
Run	1	2	3
k	10.69	33.60	13.30
n	1.26	0.93	1.29

2.2.4 HOPO

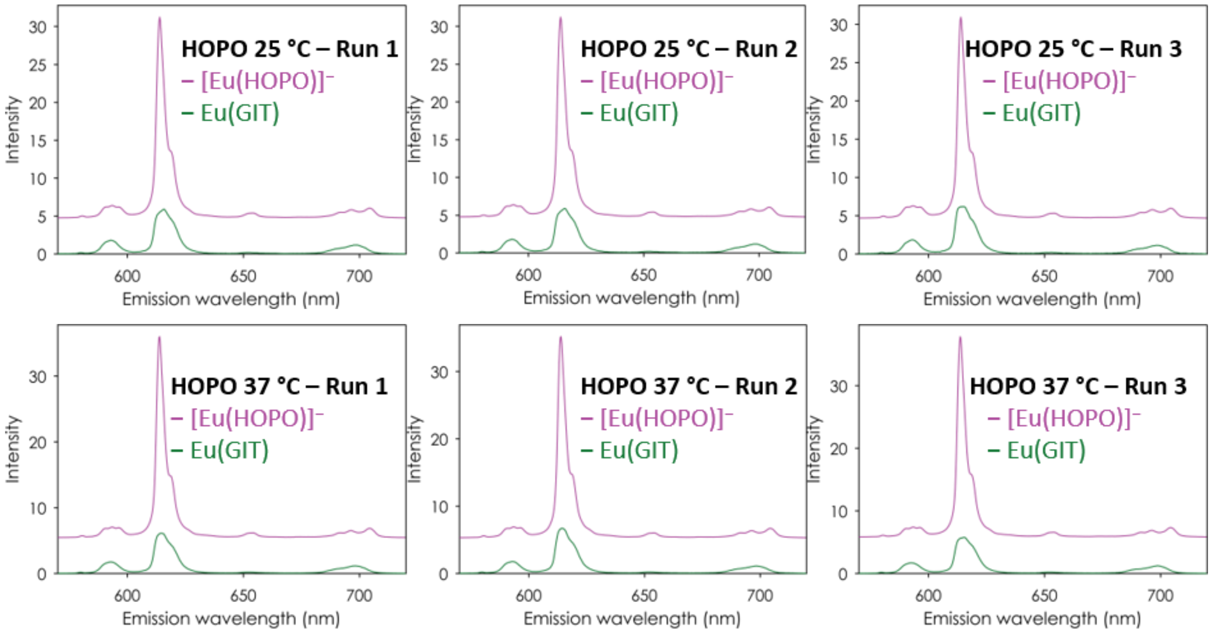

Figure S16: Emission spectra of $[Eu(HOPO)]^-$ and species formed between Eu(III) and components of GIT extracted from three different experiments at varying HOPO concentrations using PARAFAC. *T* = 25 °C (top row) or 37 °C (bottom row), [Eu(III)] = 10 µM, pH = 6.5 ± 0.5, complete GIT.

Table S15: Excitation decay lifetimes of $[Eu(HOPO)]^-$ and species formed between Eu(III) and components of GIT extracted from three different experiments at varying HOPO concentrations using PARAFAC. *T* = 25 °C (top row) or 37 °C (bottom row), [Eu(III)] = 10 µM, pH = 6.5 ± 0.5, complete GIT.

**25 °C**			
Run	1	2	3
lifetime (µs) ($[Eu(HOPO)]^-$)	669 ± 2	669 ± 2	674 ± 2
lifetime (µs) (Eu(GIT))	367 ± 10	367 ± 10	331d ± 5
**37 °C**			
Run	1	2	3
lifetime (µs) ($[Eu(HOPO)]^-$)	684 ± 3	664 ± 4	687 ± 8
lifetime (µs) (Eu(GIT))	336 ± 5	338 ± 5	343 ± 2

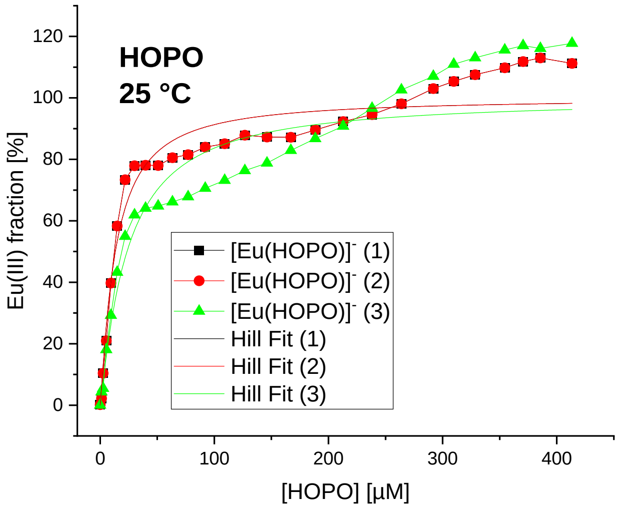

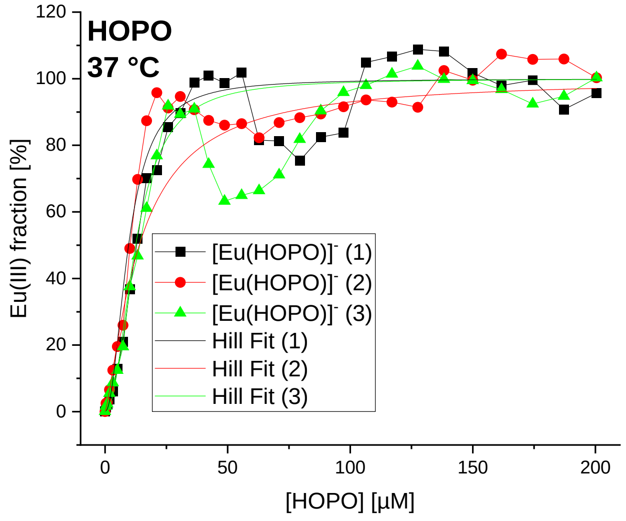

Figure S17: Fractions of the $[Eu(HOPO)]^-$ complex as function of the HOPO concentration and the corresponding Hill fits.

Table S16: Parameters obtained from Hill fit for HOPO at 25 °C and 37 °C

**25 °C**			
Run	1	2	3
k	13.33	13.33	23.56
n	1.17	1.17	1.13
**37 °C**			
Run	1	2	3
k	13.98	9.60	12.50
n	1.32	2.06	2.15

2.2.5 DOTA

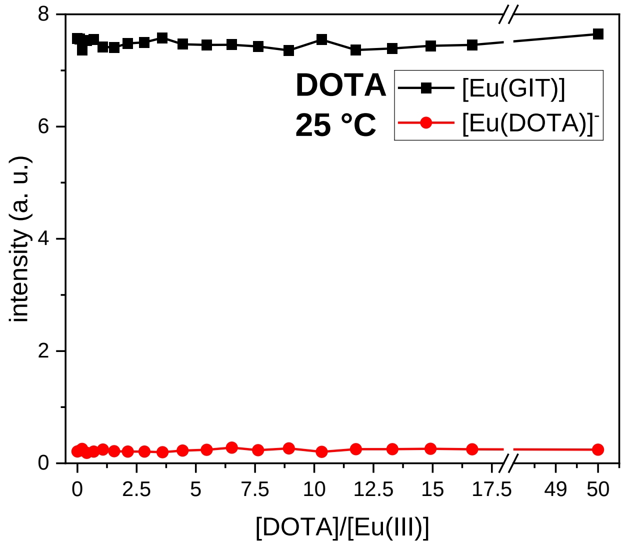

Figure S18: Emission intensity-based distribution of $[Eu(DOTA)]^-$ and Eu(III) (10 μM) bound to bioligands of the GIT mixture as a function of the DOTA/Eu(III) ratio.

2.2.6 Curium

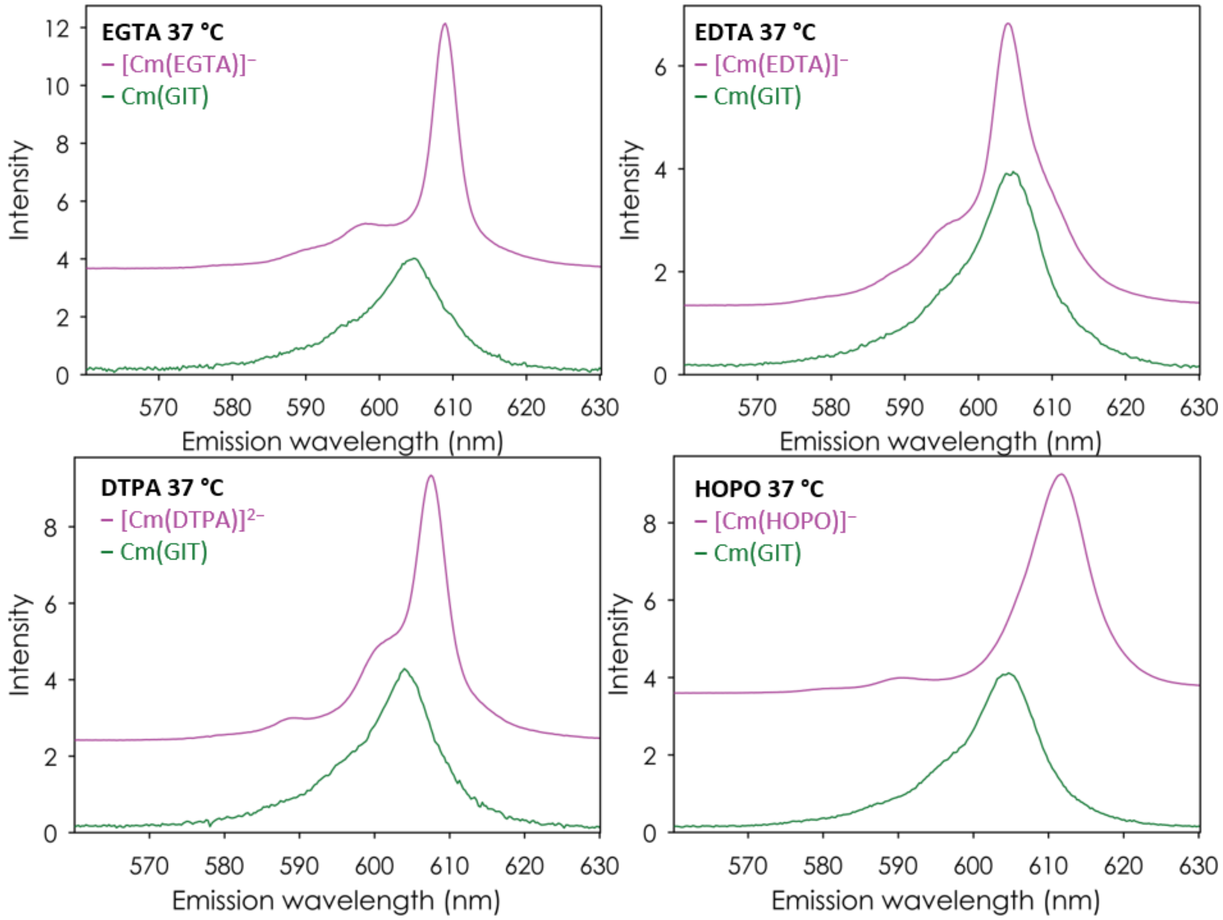

Figure S19: Emission spectra of the complexes formed between Cm(III) and EGTA, EDTA, DTPA, HOPO, and the components of GIT extracted from one experiment at varying ligand concentrations using PARAFAC. [Cm(III)] = 0.3 μM, pH = 6.5 ± 0.5, complete GIT, T = 37 °C.

3 Thermodynamic modelling

3.1.1 EGTA

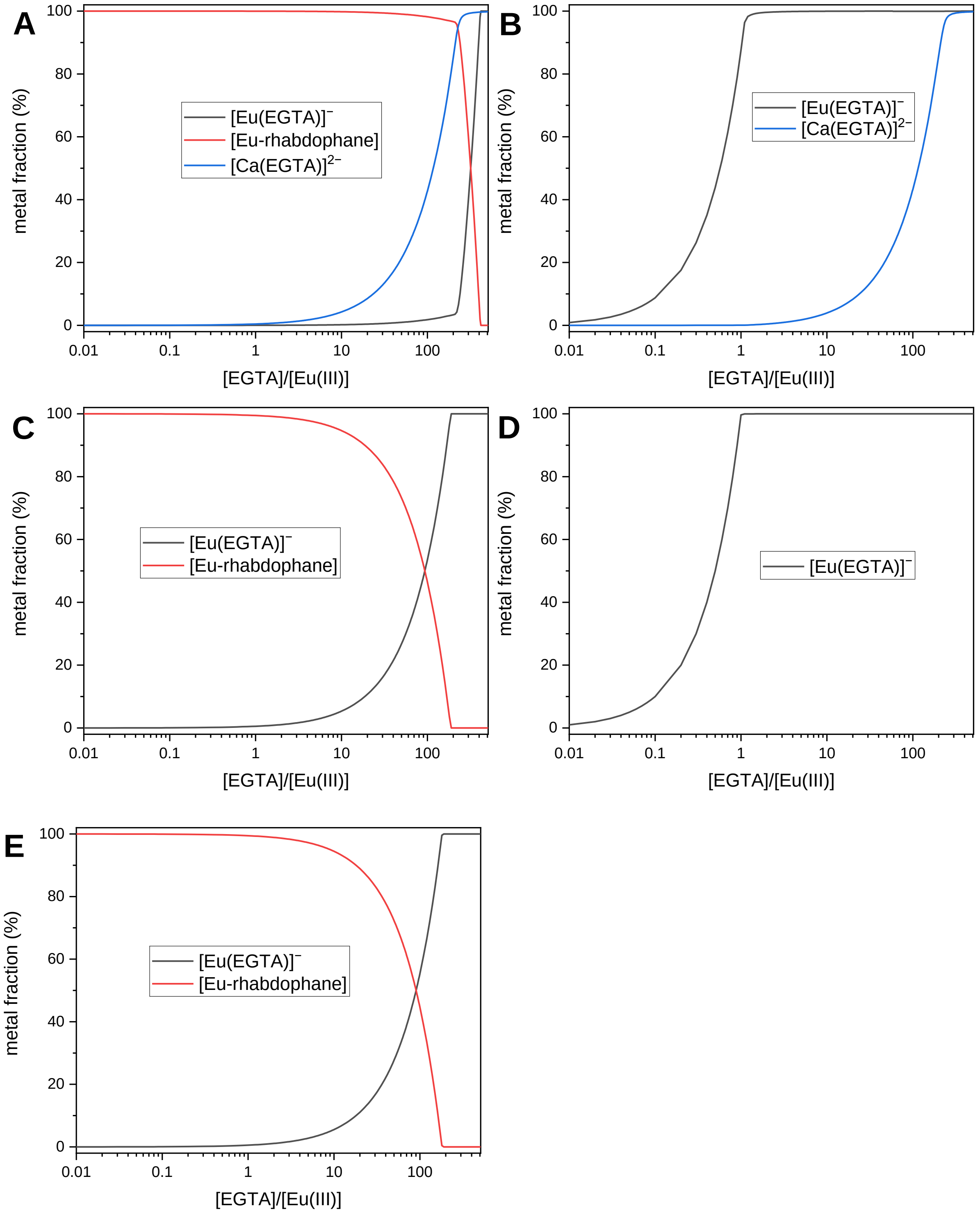

Figure S20: Metal fractions of Eu(III) and Ca(II) in dependency of the EGTA/Eu(III) ratio calculated using PHREEQC at different conditions. **A**: all inorganics of the GIT, **B**: all inorganics of the GIT except phosphate, **C**: all inorganics of the GIT except calcium, **D**: all inorganics of the GIT except phosphate and calcium, **E**: only the phosphate fraction (3.73 mM) and the ionic strength (344 mM) of the GIT. [Eu(III)] = 10 µM, pH = 6.5.

Table S17: Calculated DC_{50} values of EGTA using PHREEQC at different conditions.

**condition**	DC_{50} (×[Eu(III)])
full GIT inorganics (**A**)	320
GIT inorganics without phosphate (**B**)	0.57
GIT inorganics without Ca(II) (**C**)	93.4
GIT inorganics without phosphate and Ca(II) (**D**)	0.50
Only GIT phosphate fraction (**E**)	90.3

3.1.2 EDTA

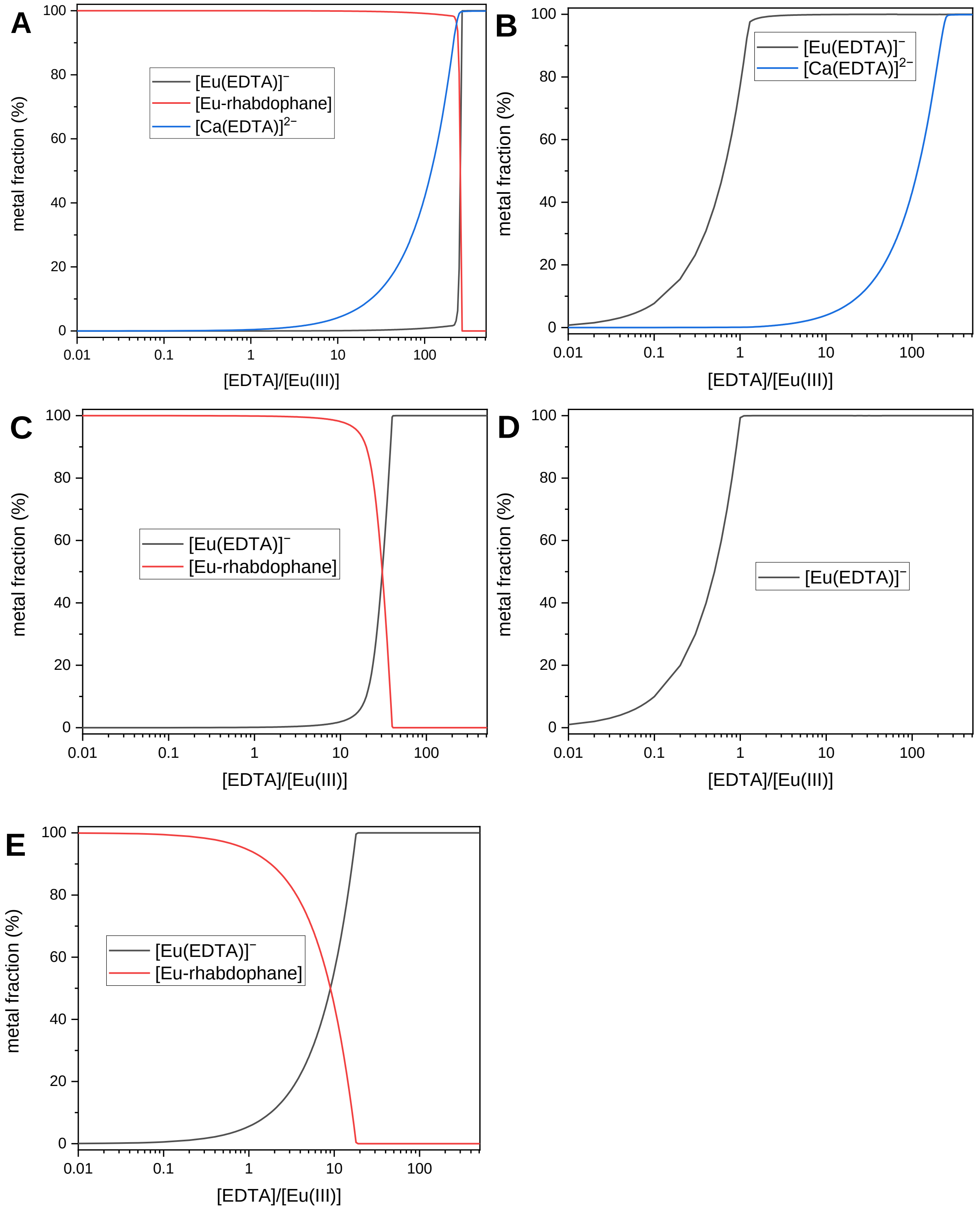

Figure S21: Metal fractions of Eu(III) and Ca(II) in dependency of the EDTA/Eu(III) ratio calculated using PHREEQC at different conditions. **A**: all inorganics of the GIT, **B**: all inorganics of the GIT except phosphate, **C**: all inorganics of the GIT except calcium, **D**: all inorganics of the GIT except phosphate and calcium, **E**: only the phosphate fraction (3.73 mM) and the ionic strength (344 mM) of the GIT. [Eu(III)] = 10 μM, pH = 6.5.

Table S18: Calculated DC_{50} values of EDTA using PHREEQC at different conditions.

**condition**	DC_{50} (×[Eu(III)])
full GIT inorganics (**A**)	258
GIT inorganics without phosphate (**B**)	0.65
GIT inorganics without Ca(II) (**C**)	30.6
GIT inorganics without phosphate and Ca(II) (**D**)	0.50
Only GIT phosphate fraction (**E**)	9.02

3.1.3 DTPA

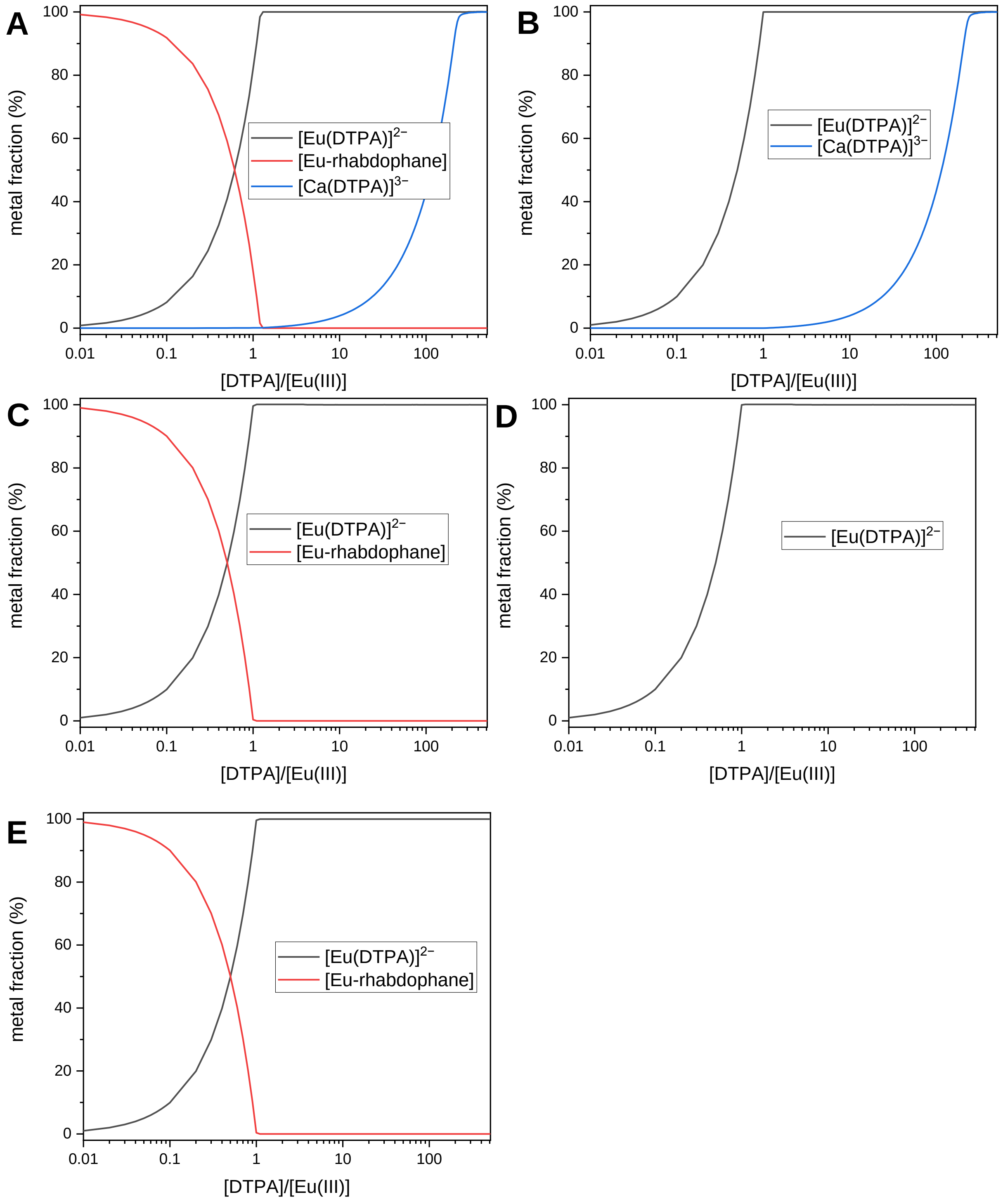

Figure S22: Metal fractions of Eu(III) and Ca(II) in dependency of the DTPA/Eu(III) ratio calculated using PHREEQC at different conditions. **A**: all inorganics of the GIT, **B**: all inorganics of the GIT except phosphate, **C**: all inorganics of the GIT except calcium, **D**: all inorganics of the GIT except phosphate and calcium, **E**: only the phosphate fraction (3.73 mM) and the ionic strength (344 mM) of the GIT. [Eu(III)] = 10 µM, pH = 6.5.

Table S19: Calculated DC_{50} values of DTPA using PHREEQC at different conditions.

**condition**	DC_{50} (×[Eu(III)])
full GIT inorganics (**A**)	0.61
GIT inorganics without phosphate (**B**)	0.50
GIT inorganics without Ca(II) (**C**)	0.50
GIT inorganics without phosphate and Ca(II) (**D**)	0.50
Only GIT phosphate fraction (**E**)	0.50

3.1.4 HOPO

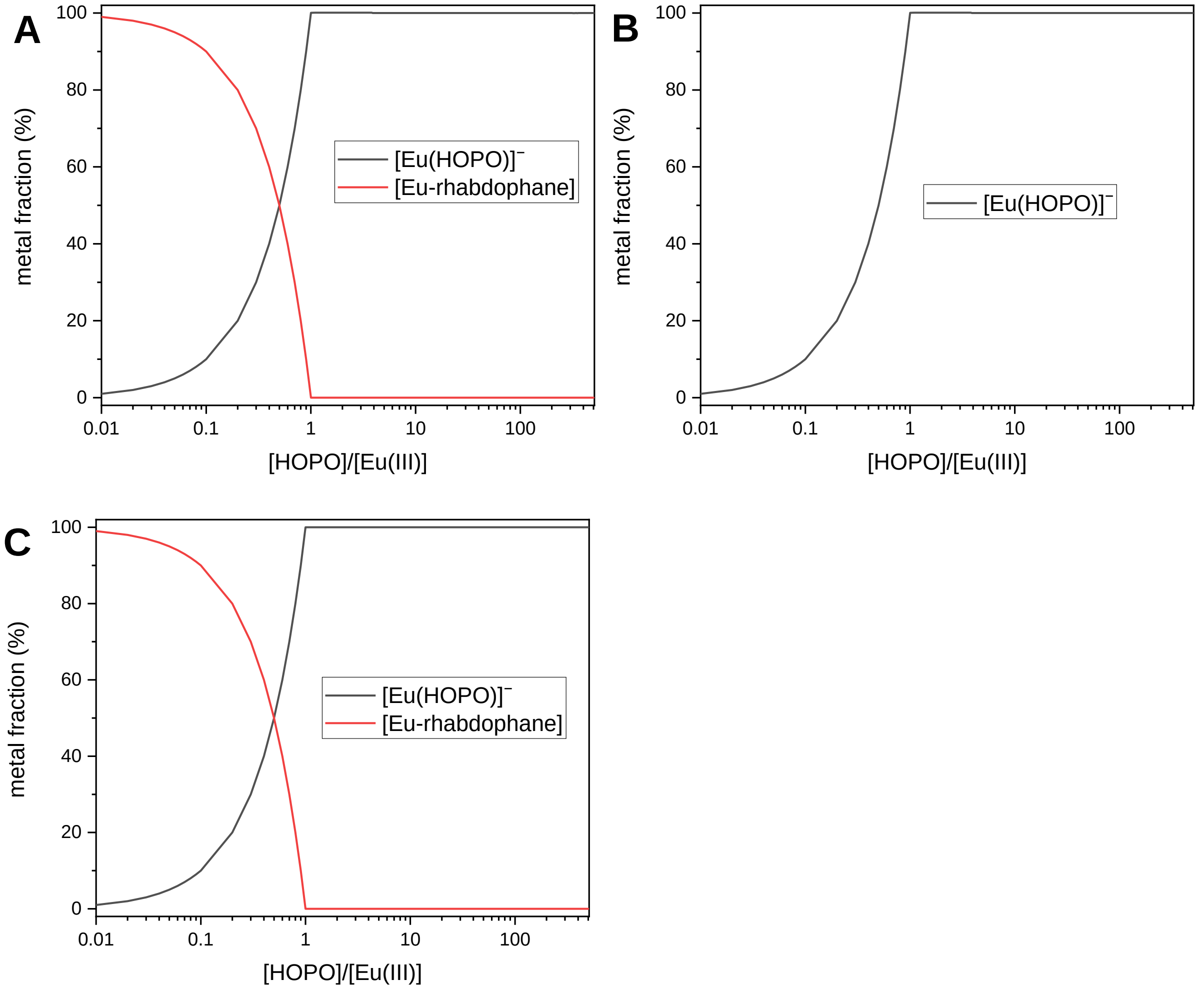

Figure S23: Metal fractions of Eu(III) in dependency of the HOPO/Eu(III) ratio calculated using PHREEQC at different conditions. **A**: all inorganics of the GIT except calcium, **B**: all inorganics of the GIT except phosphate and calcium, **C**: only the phosphate fraction (3.73 mM) and the ionic strength (344 mM) of the GIT. [Eu(III)] = 10 µM, pH = 6.5.

Table S20: Calculated DC_{50} values of HOPO using PHREEQC at different conditions.

**condition**	DC_{50} (×[Eu(III)])
GIT inorganics without Ca(II) (**A**)	0.50
GIT inorganics without phosphate and Ca(II) (**B**)	0.50
Only GIT phosphate fraction (**C**)	0.50

3.1.5 DOTA

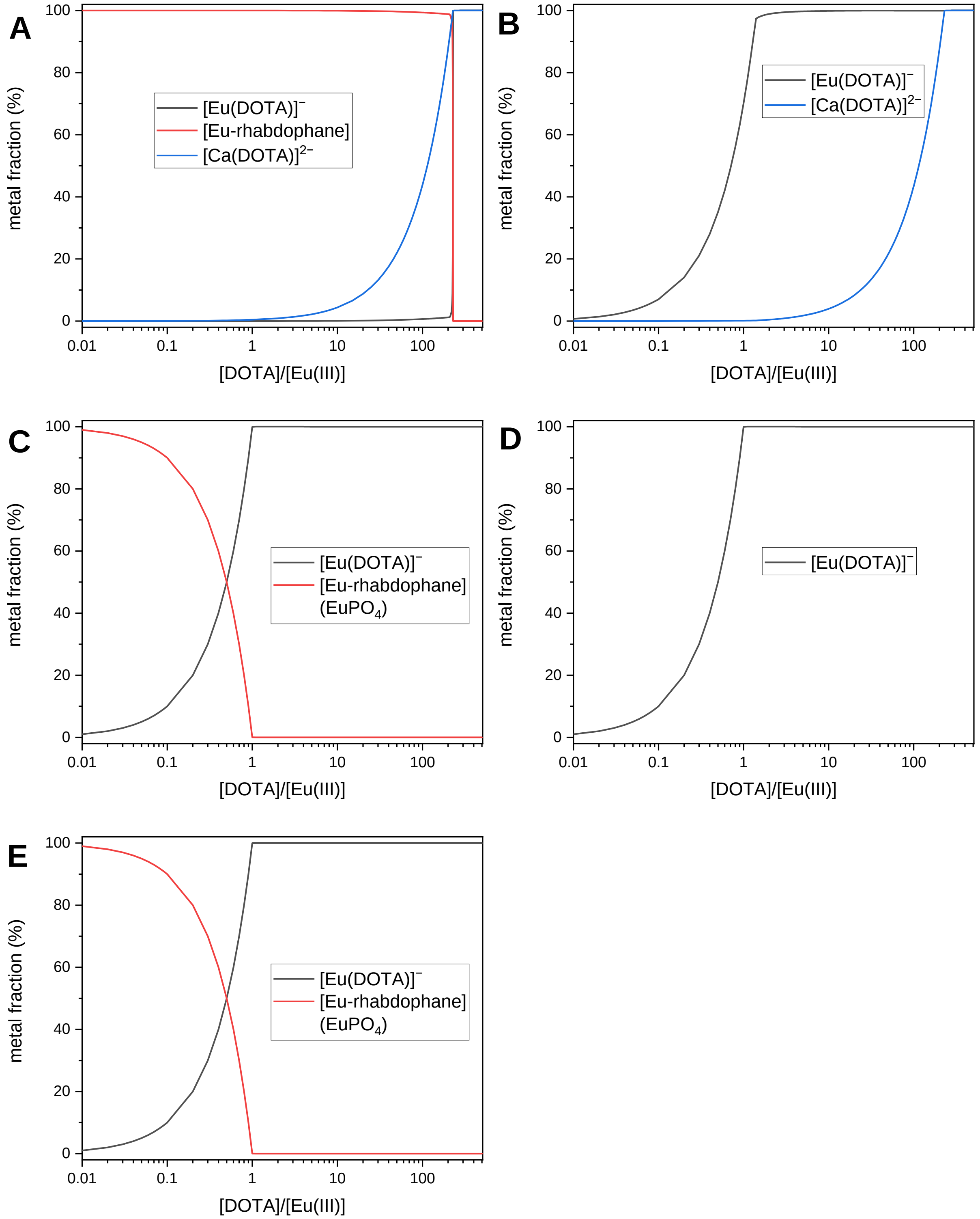

Figure S24: Metal fractions of Eu(III) and Ca(II) in dependency of the DOTA/Eu(III) ratio calculated using PHREEQC at different conditions. **A**: all inorganics of the GIT, **B**: all inorganics of the GIT except phosphate, **C**: all inorganics of the GIT except calcium, **D**: all inorganics of the GIT except phosphate and calcium, **E**: only the phosphate fraction (3.73 mM) and the ionic strength (344 mM) of the GIT. [Eu(III)] = 10 µM, pH = 6.5.

Table S21: Calculated DC_{50} values of DOTA using PHREEQC at different conditions.

**condition**	DC_{50} (×[Eu(III)])
full GIT inorganics (**A**)	225
GIT inorganics without phosphate (**B**)	0.71
GIT inorganics without Ca(II) (**C**)	0.50
GIT inorganics without phosphate and Ca(II) (**D**)	0.50
Only GIT phosphate fraction (**E**)	0.50

3.1.6 DEGTA

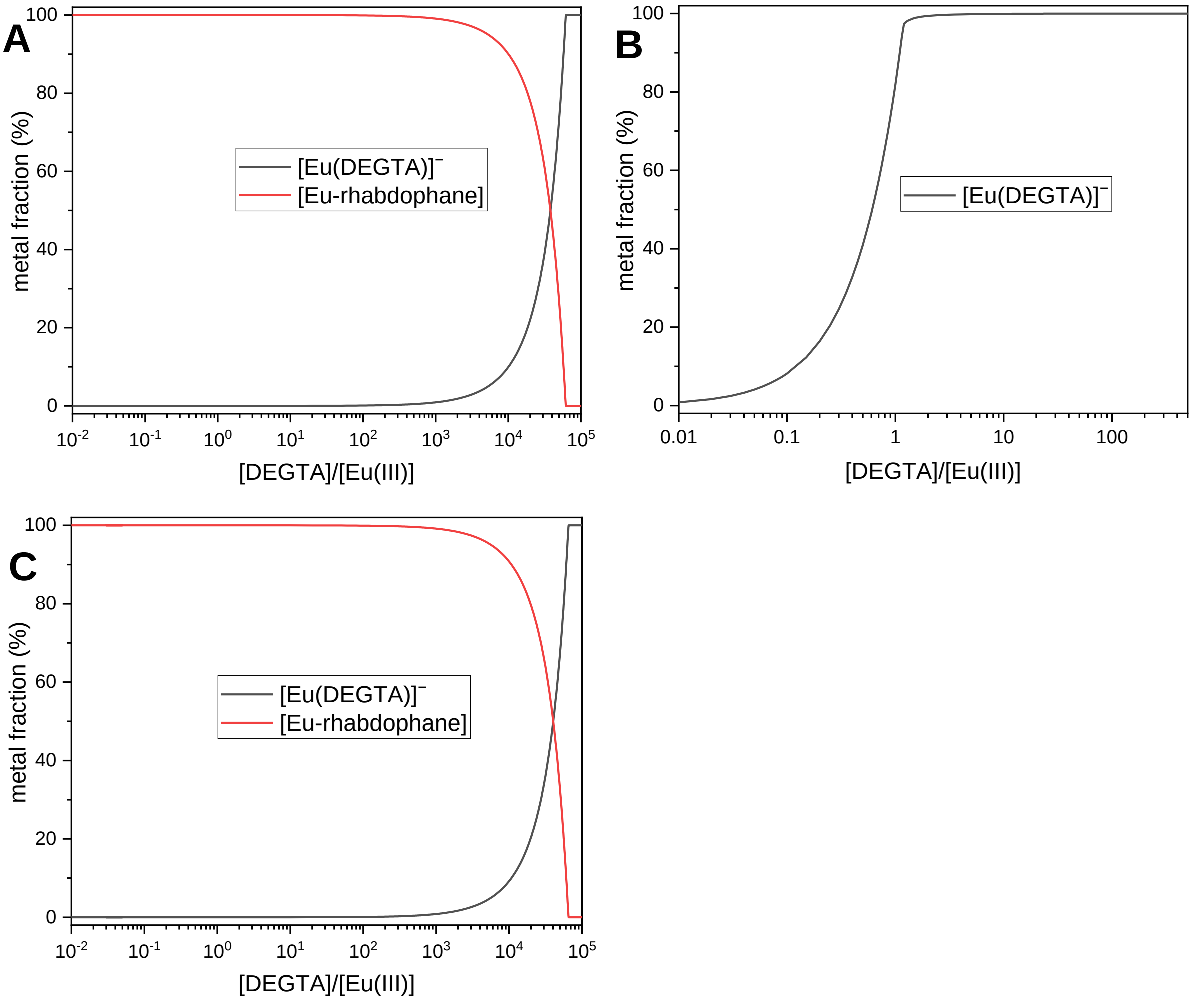

Figure S25: Metal fractions of Eu(III) in dependency of the DEGTA/Eu(III) ratio calculated using PHREEQC at different conditions. **A**: all inorganics of the GIT except calcium, **B**: all inorganics of the GIT except phosphate and calcium, **C**: only the phosphate fraction (3.73 mM) and the ionic strength (344 mM) of the GIT. [Eu(III)] = 10 µM, pH = 6.5.

Table S22: Calculated DC_{50} values of DEGTA using PHREEQC at different conditions.

**condition**	DC_{50} (×[Eu(III)])
GIT inorganics without Ca(II) (**A**)	38048
GIT inorganics without phosphate and Ca(II) (**B**)	0.61
Only GIT phosphate fraction (**C**)	40311

4 ^{2}H-NMR spectroscopy

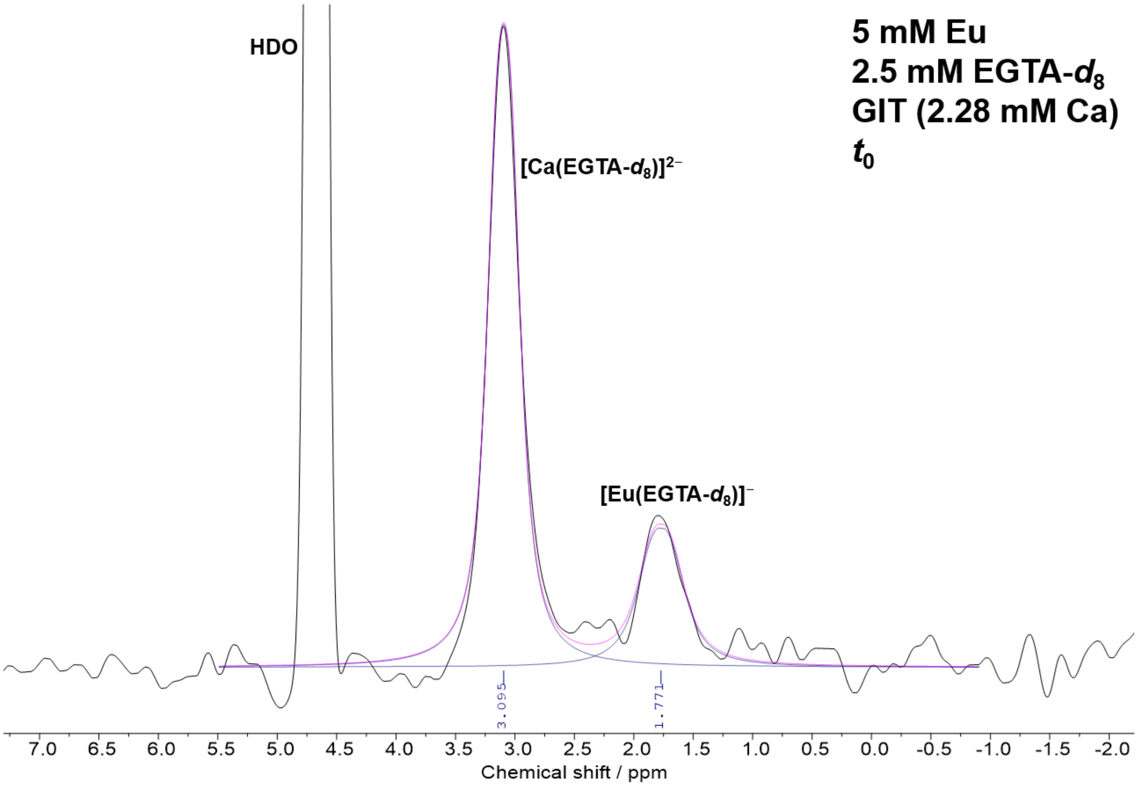

Figure S26: ^{2}H-NMR spectra of Eu(III) and EGTA-d_8 in the full GIT solution taken immediately after preparation. The signals of Eu(III)- and Ca(II)-bound EGTA-d_8 are deconvoluted. [Eu(III)] = 5 mM, [EGTA-d_8] = 2.5 mM.

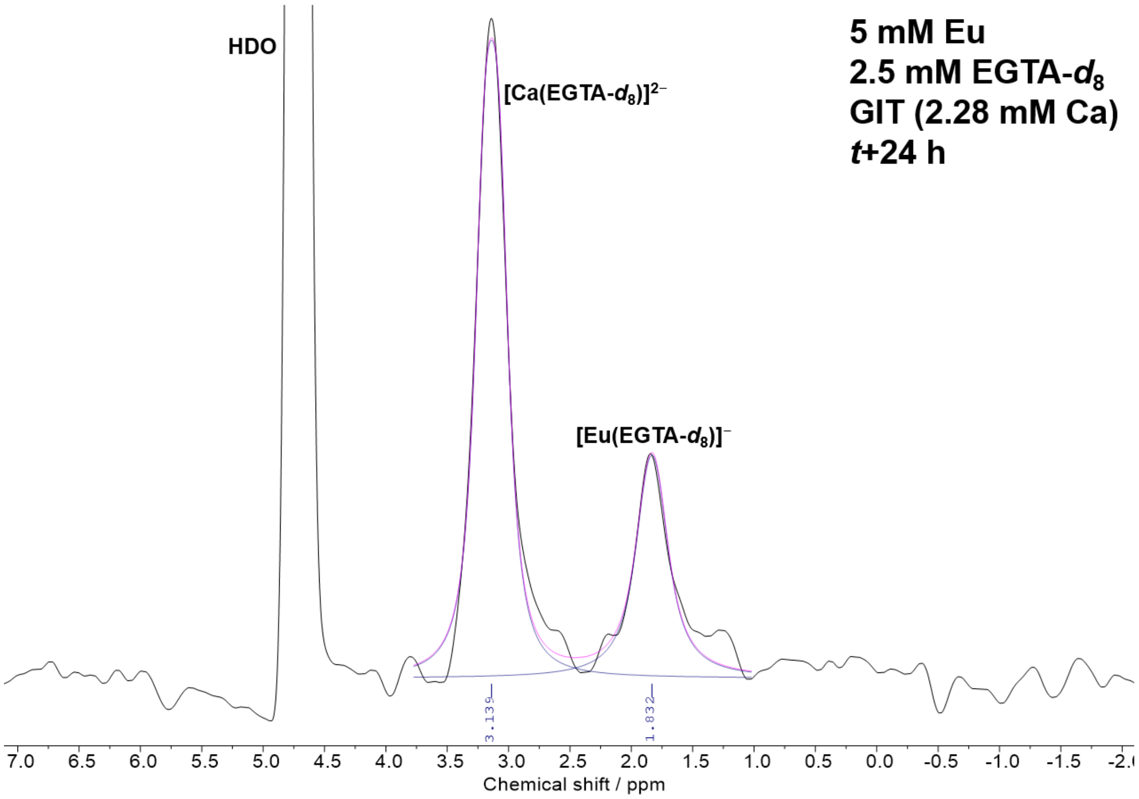

Figure S27: ^{2}H-NMR spectra of Eu(III) and EGTA-d_8 in the full GIT solution taken 24 hours after preparation. The signals of Eu(III)- and Ca(II)-bound EGTA-d_8 are deconvoluted. [Eu(III)] = 5 mM, [EGTA-d_8] = 2.5 mM.

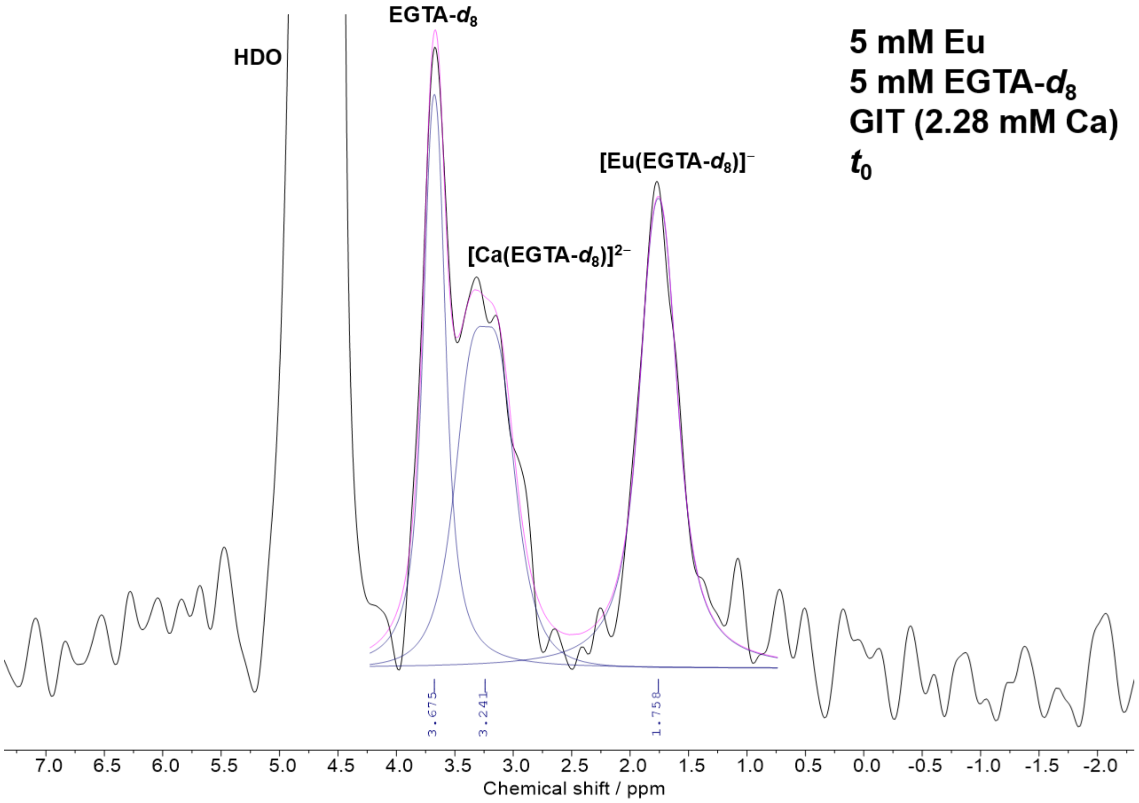

Figure S28: ^{2}H-NMR spectra of Eu(III) and EGTA-*d*8 in the full GIT solution taken immediately after preparation. The signals of Eu(III)- and Ca(II)-bound EGTA-*d*8 are deconvoluted. [Eu(III)] = 5 mM, [EGTA-*d*8] = 5 mM.

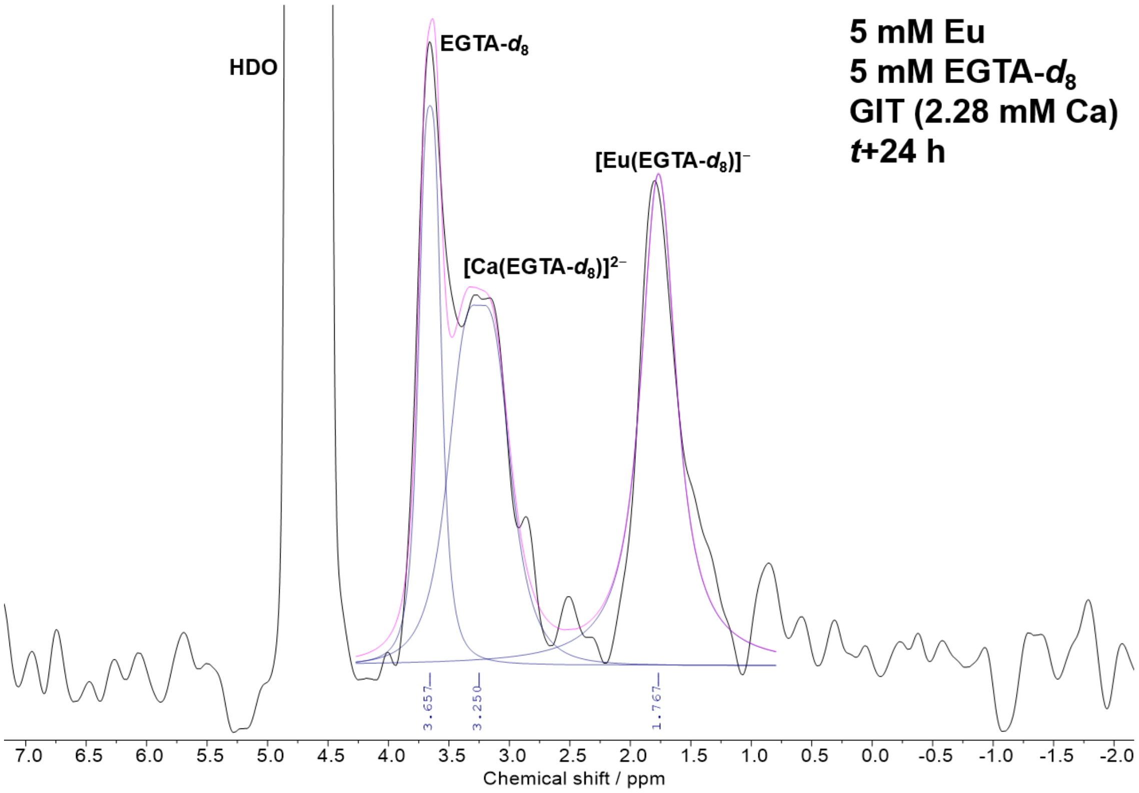

Figure S29: ^{2}H-NMR spectra of Eu(III) and EGTA-*d*8 in the full GIT solution taken 24 hours after preparation. The signals of free, Eu(III)- and Ca(II)-bound EGTA-*d*8 are deconvoluted. [Eu(III)] = 5 mM, [EGTA-*d*8] = 5 mM.

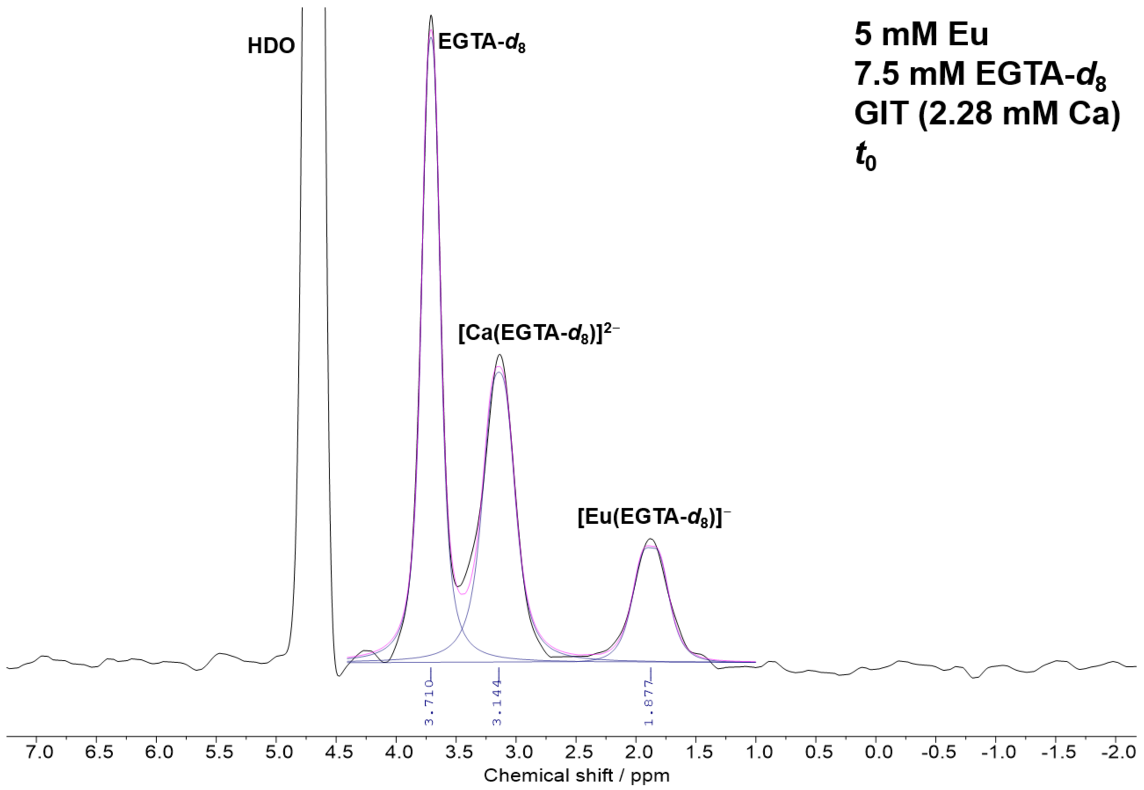

Figure S30: ^{2}H-NMR spectra of Eu(III) and EGTA-d_8 in the full GIT solution taken immediately after preparation. The signals of free, Eu(III)- and Ca(II)-bound EGTA-d_8 are deconvoluted. [Eu(III)] = 5 mM, [EGTA-d_8] = 7.5 mM.

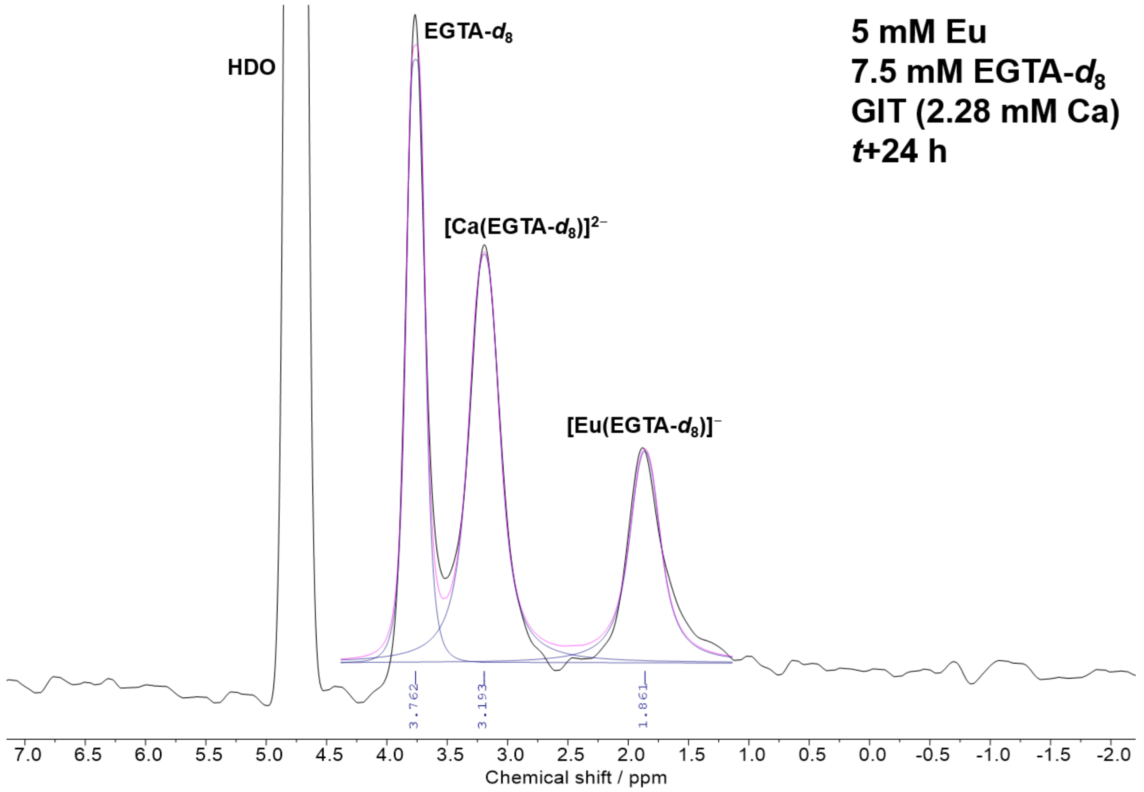

Figure S31: ^{2}H-NMR spectra of Eu(III) and EGTA-d_8 in the full GIT solution taken 24 hours after preparation. The signals of free, Eu(III)- and Ca(II)-bound EGTA-d_8 are deconvoluted. [Eu(III)] = 5 mM, [EGTA-d_8] = 7.5 mM.

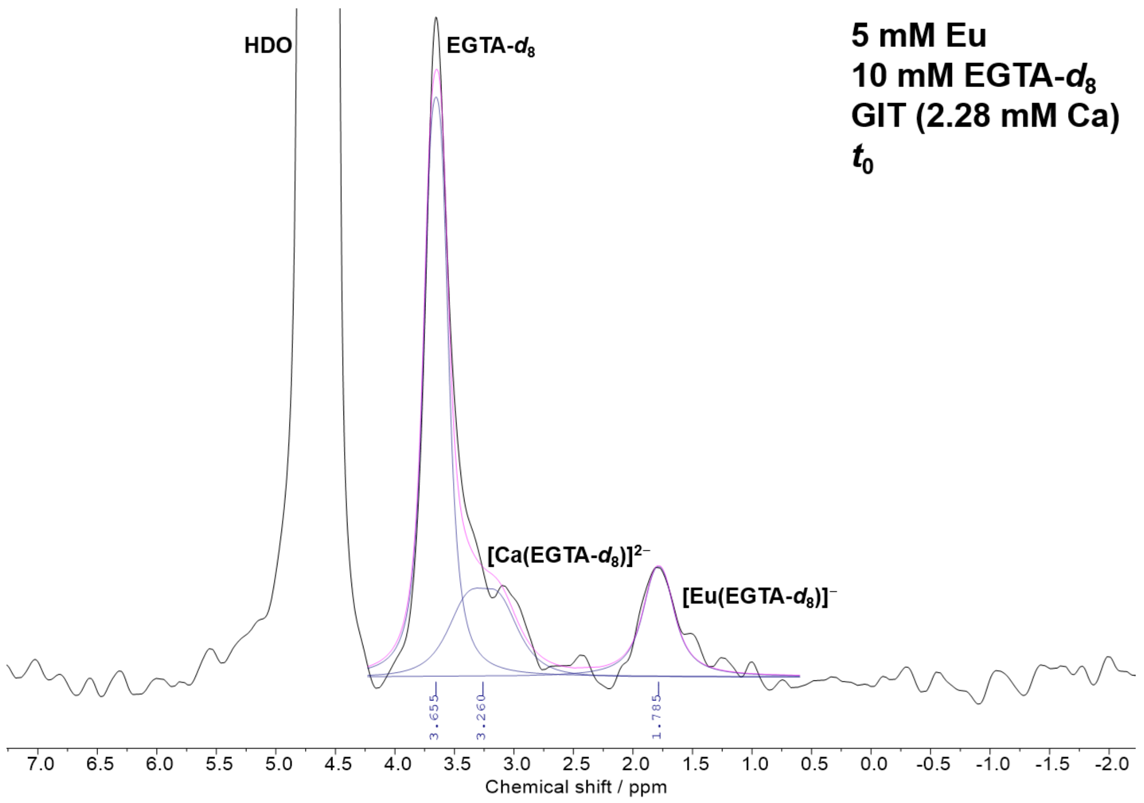

Figure S32: ^{2}H-NMR spectra of Eu(III) and EGTA-*d*$_8$ in the full GIT solution taken immediately after preparation. The signals of free, Eu(III)- and Ca(II)-bound EGTA-*d*$_8$ are deconvoluted. [Eu(III)] = 5 mM, [EGTA-*d*$_8$] = 10 mM.

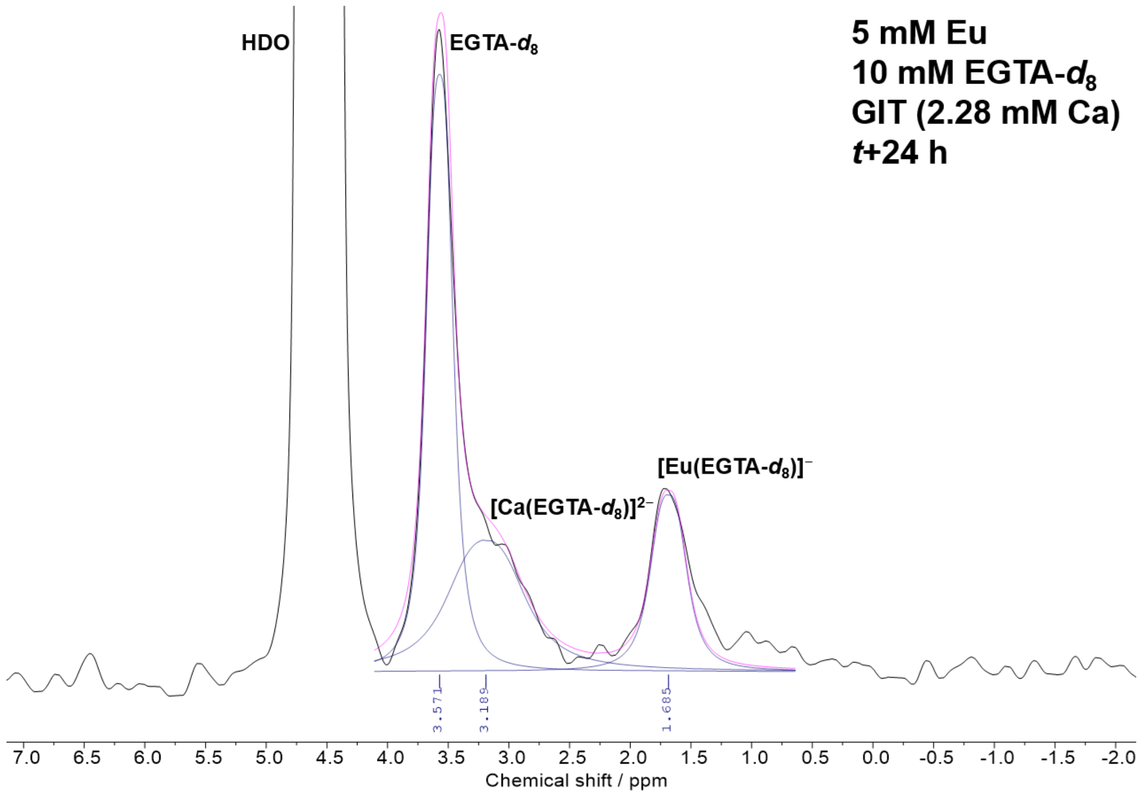

Figure S33: ^{2}H-NMR spectra of Eu(III) and EGTA-*d*$_8$ in the full GIT solution taken 24 hours after preparation. The signals of free, Eu(III)- and Ca(II)-bound EGTA-*d*$_8$ are deconvoluted. [Eu(III)] = 5 mM, [EGTA-*d*$_8$] = 10 mM.

Table S23: Areas of the signals of free, Eu(III)- and Ca(II)-bound EGTA-d_8 of Eu(III) and EGTA-d_8 in the full GIT at varying EGTA-d_8 concentrations and time points obtained by ^{2}H-NMR spectroscopy.

[EGTA-d_8] (mM)	Time (h)	Area (a. u.)		
		Free [EGTA-d_8]	$[Ca(EGTA\text{-}d_8)]^{2-}$	$[Eu(EGTA\text{-}d_8)]^{-}$
2.5	0	-	3654.15	1162.59
	24	-	4342.34	1902.64
5	0	677.19	809.66	943.01
	24	673.82	1089.39	1238.66
7.5	0	7737.44	5931.27	2331.18
	24	5244.02	6934.53	3823.43
10	0	1648.92	598.94	466.90
	24	2856.85	1959.78	1176.97

5 Photographs

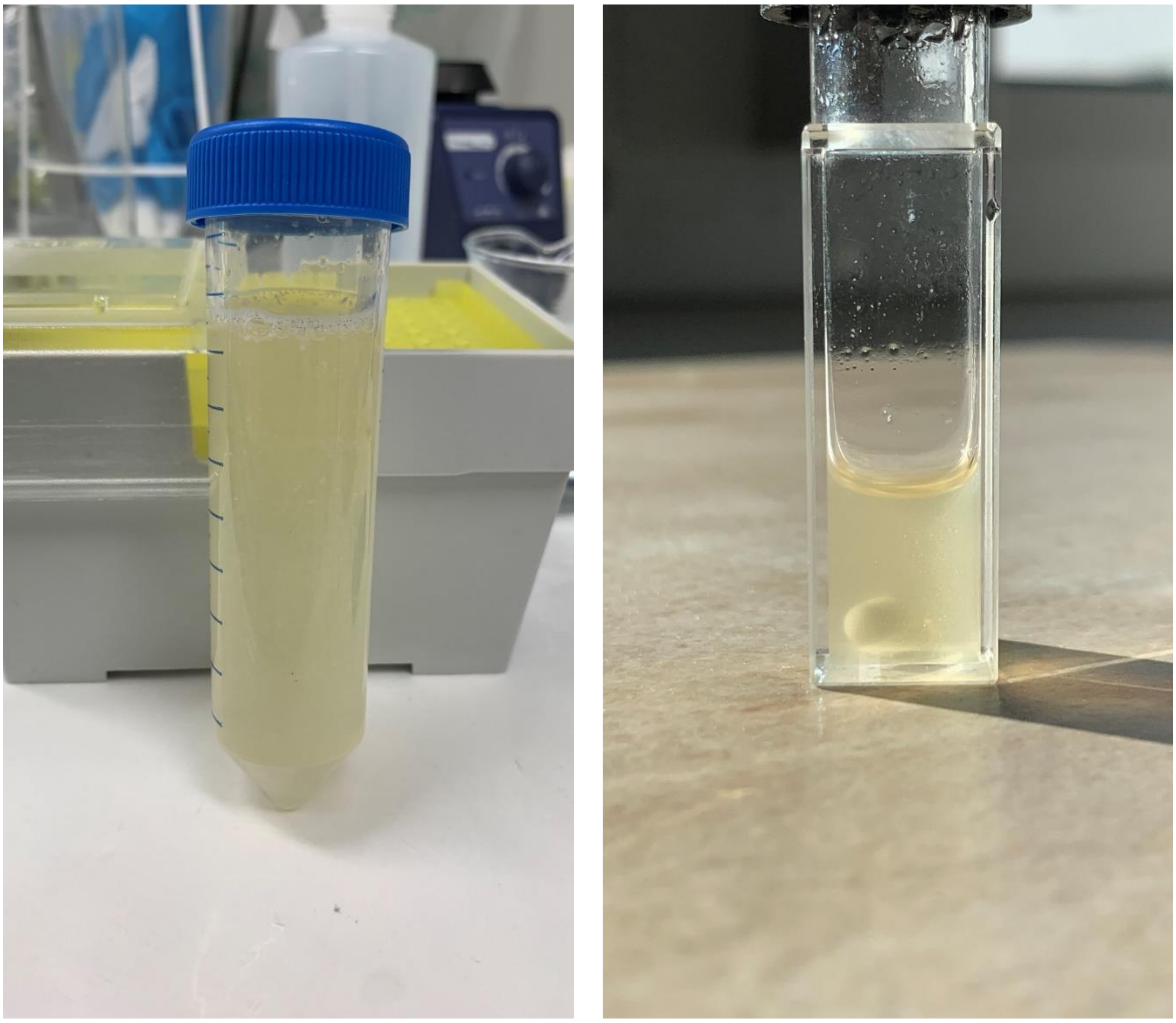

Figure S34: Photographs of the GIT solutions. Left: after preparation and equilibration of the artificial GIT solution. Right: GIT solution containing 1 mM of HOPO after measuring at 37 °C. [Eu(III)] = 10 µM, pH = 6.5 ± 0.5.
